# Tumor suppressor microRNAs are downregulated in myelodysplastic syndrome with spliceosome mutations

**DOI:** 10.18632/oncotarget.7127

**Published:** 2016-02-02

**Authors:** Derya Aslan, Christian Garde, Mette Katrine Nygaard, Alexandra Søgaard Helbo, Konstantinos Dimopoulos, Jakob Werner Hansen, Marianne Tang Severinsen, Marianne Bach Treppendahl, Lene Dissing Sjø, Kirsten Grønbæk, Lasse Sommer Kristensen

**Affiliations:** ^1^ Department of Hematology, Rigshospitalet, Copenhagen, Denmark; ^2^ Center for Biological Sequence Analysis, Department of Systems Biology, Technical University of Denmark, Kgs. Lyngby, Denmark; ^3^ Department of Hematology, Aalborg University Hospital, Aalborg, Denmark; ^4^ Department of Pathology, Rigshospitalet, Copenhagen, Denmark

**Keywords:** myelodysplastic syndrome, microRNAs, spliceosome mutations, RT-qPCR, high-resolution melting

## Abstract

Spliceosome mutations are frequently observed in patients with myelodysplastic syndromes (MDS). However, it is largely unknown how these mutations contribute to the disease. MicroRNAs (miRNAs) are small noncoding RNAs, which have been implicated in most human cancers due to their role in post transcriptional gene regulation. The aim of this study was to analyze the impact of spliceosome mutations on the expression of miRNAs in a cohort of 34 MDS patients. In total, the expression of 76 miRNAs, including mirtrons and splice site overlapping miRNAs, was accurately quantified using reverse transcriptase quantitative PCR. The majority of the studied miRNAs have previously been implicated in MDS. Stably expressed miRNA genes for normalization of the data were identified using GeNorm and NormFinder algorithms. High-resolution melting assays covering all mutational hotspots within *SF3B1*, *SRSF2*, and *U2AF1* (*U2AF35*) were developed, and all detected mutations were confirmed by Sanger sequencing. Overall, canonical miRNAs were downregulated in spliceosome mutated samples compared to wild-type (*P* = 0.002), and samples from spliceosome mutated patients clustered together in hierarchical cluster analyses. Among the most downregulated miRNAs were several tumor-suppressor miRNAs, including several let-7 family members, miR-423, and miR-103a. Finally, we observed that the predicted targets of the most downregulated miRNAs were involved in apoptosis, hematopoiesis, and acute myeloid leukemia among other cancer- and metabolic pathways. Our data indicate that spliceosome mutations may play an important role in MDS pathophysiology by affecting the expression of tumor suppressor miRNA genes involved in the development and progression of MDS.

## INTRODUCTION

Myelodysplastic syndromes (MDS) comprise a diverse group of clonal hematopoietic stem cell malignancies characterized by dysplasia, peripheral cytopenias, ineffective hematopoiesis, and risk of progression to acute myeloid leukemia (AML). It is the common understanding that MDS originates from a founding driver mutation within a cell having the capacity to differentiate into mature myeloid cells [1]. The nature of the founding mutation is likely to be involved in determining clinical phenotype and outcome. For instance, mutations in *SF3B1* are strongly associated with refractory anemia with ring sideroblasts (RARS) and a good prognosis [2, 3], whereas *SRSF2* and *U2AF1* (*U2AF35*) mutations are mainly observed in refractory cytopenia with multi lineage dysplasia (RCMD) and refractory anemia with excess blasts (RAEB), and associated with high risk of leukemic transformation [4].

The founding mutation will eventually be present in almost all bone marrow cells due to the selective advantage gained by the cell in which the mutation occurred. These clonal cells also carry hundreds of benign somatic passenger mutations captured at the start of clonal proliferation [5]. Most often the driver mutations are found in genes involved in RNA splicing, such as *SF3B1*, *SRSF2*, *U2AF35*, and *ZRSR2* or in epigenetic regulation, such as *TET2*, *DNMT3A*, *IDH1/2*, *ASXL1*, and *EZH2* [6–8]. As opposed to mutations in pro-apoptotic genes and growth signaling pathways these mutations do not, by themselves, promote clonal proliferation. However, they may affect the expression of a large number of downstream protein-coding- and microRNA (miRNAs) genes, which lead to expansion of the mutated clone.

In addition to the genetic alterations observed in MDS, several miRNAs have been found to be aberrantly expressed in this disease [9]. Most miRNAs are processed through the canonical pathway involving the nuclease Drosha and its cofactor DGCR8. These canonical miRNAs may be located in intergenic regions or located within introns and exons of protein-coding genes and share their regulatory elements. However, some miRNAs are transcribed from the opposite strand in the reverse direction of the gene, which may lead to an inverse correlation between the expression of the gene and the miRNA [10]. In addition, an emerging number of miRNAs have recently been shown to bypass Drosha by utilizing the spliceosome instead. These are located within introns of protein encoding genes and are known as mirtrons when the 5′ and 3′ ends of the precursor miRNA transcript is defined by splice sites, and tailed mirtrons when either the 5′ or the 3′ end is defined by a splice site [11–13]. Thus, tailed mirtrons are processed by both splicing and exonuclease digestion. Finally, a competitive interaction between the splicing machinery and the Drosha/DGCR8 complex has been discovered for miRNAs with precursors that are splice site overlapping (SO miRNAs) [14, 15].

Here, we studied the effects of spliceosome mutations on miRNA expression in primary bone marrow samples from MDS patients. To avoid potential confounding effects only patients with refractory anemia RA with or without ringsideroblasts were included in the study. High-resolution melting (HRM) assays covering all mutational hotspots of *SF3B1*, *SRSF2*, and *U2AF35* were developed. All mutations detected by HRM in the patient samples were subsequently confirmed by Sanger sequencing. In total, 76 miRNAs were studied, including intragenic and intergenic canonical miRNAs previously implicated in MDS, as well as mirtrons, tailed mirtrons, and SO miRNAs.

## RESULTS

### Spliceosome mutations in the patient samples

**Figure 1:**
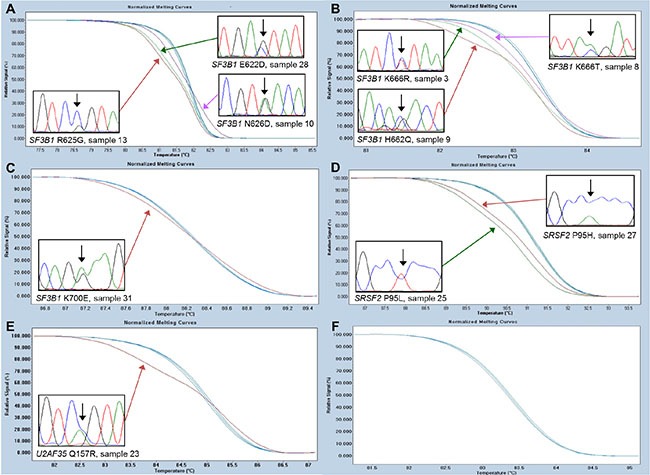
High-resolution melting assays for the detection of SF3B1, SRSF2, and U2AF35 mutations Representative data for each of the hotspots are shown. (**A**) *SF3B1* hotspot containing the N626D, R625G, and E622D detected in sample 10, 13, and 28, respectively. (**B**) *SF3B1* hotspot containing the K666R, K666T, and H662Q mutations detected in sample 3, 8, and 9, respectively. (**C**) *SF3B1* hotspot containing the K700E mutation detected in sample 31. (**D**) *SRSF2* hotspot containing the P95L and P95H mutations detected in sample 25 and 27, respectively. (**E**) *U2AF35* hotspot containing the Q157R mutation detected in sample 23. (**F**) *U2AF35* hotspot containing the S34 mutations not detected in any of the samples.

**Figure 2:**
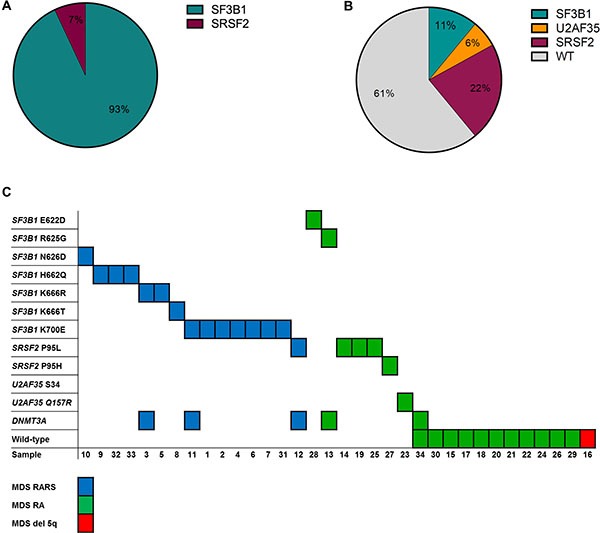
Overview of the spliceosome and *DNMT3A* mutations detected in the MDS samples (**A**) Distribution of the mutations detected in the RARS patients. (**B**) Distribution of the mutations detected in the RA and del 5q patient samples. (**C**) Number and nature of the mutations detected in individual patients of different subtype.

All patients were screened for mutations within *SF3B1*, *SRSF2*, and *U2AF35* using HRM analysis. Since we observed a higher frequency of *SF3B1* mutations than expected [6], the samples were also tested for mutations in *SF3B1* using denaturing gradient gel electrophoresis (DGGE) analyses (data not shown). The results were 100% concordant between the HRM and DGGE analyses, and all mutations were confirmed by Sanger sequencing (Supplementary Table S1, Figure 1). Twenty-two of the patients had a spliceosome mutation and 16 of these were found in *SF3B1*, five in *SRSF2* and one in *U2AF35*. The mutations were unevenly distributed across RARS, RA, and del 5q subtypes. Among the patients with RARS 14 out of 15 (93%) patients carried a mutation in *SF3B1* and one carried a mutation in *SRSF2* (7%). Among the patients with RA two out of 18 carried a mutation in *SF3B1* (11%), four carried a mutation in *SRSF2* (22%), and one carried a mutation in *U2AF35* (6%). All mutations were mutually exclusive (Figure 2). Because *SF3B1* mutations have a tendency to coexist with *DNMT3A* mutations [8] the samples were also screened for mutations within this gene (Supplementary Table S1, Supplementary Figure S1). In total, five of the patients carried a mutation in *DNMT3A*. Three of these patients also carried an *SF3B1* mutation and one carried a mutation in *SRSF2* (Figure 2).

### Identification of accurate reference miRNA genes for the RT-qPCR analysis

For normalization of the miRNA expression data the identification of stably expressed reference genes are of paramount importance [16]. For this purpose we used the GeNorm and NormFinder algorithms [17, 18]. Data for 29 miRNAs and three commonly used reference genes (*SNORD44*, *SNORD47*, and *RNU6B*), which were expressed in all samples, were included in the analyses. Overall, there was a striking resemblance between the data obtained by the two algorithms (Supplementary Figure S2). The potential reference genes *RNU6B*, *SNORD44*, and *SNORD47* were ranked ninth, 13th, and 22nd, respectively, by NormFinder, and 11th, 15th, and 21st, respectively, by GeNorm. Both algorithms identified miR-10b-5p and miR-486-5p as the least stable genes, while miR-106b-3p was the most stable gene in both analyses. NormFinder identified miR-29a-3p and miR-130b-3p as the best combination of any two reference genes. These two miRNAs were also among the most stably expressed genes in the GeNorm analysis (Supplementary Figure S2). As NormFinder takes into account that different biological subgroups exists within the cohort, we decided to use the geometric mean of the Ct-values for miR-29a-3p and miR-130b-3p when calculating relative expression quantities for each miRNA of interest.

### Overall influences of spliceosome mutations on miRNA expression levels

**Figure 3:**
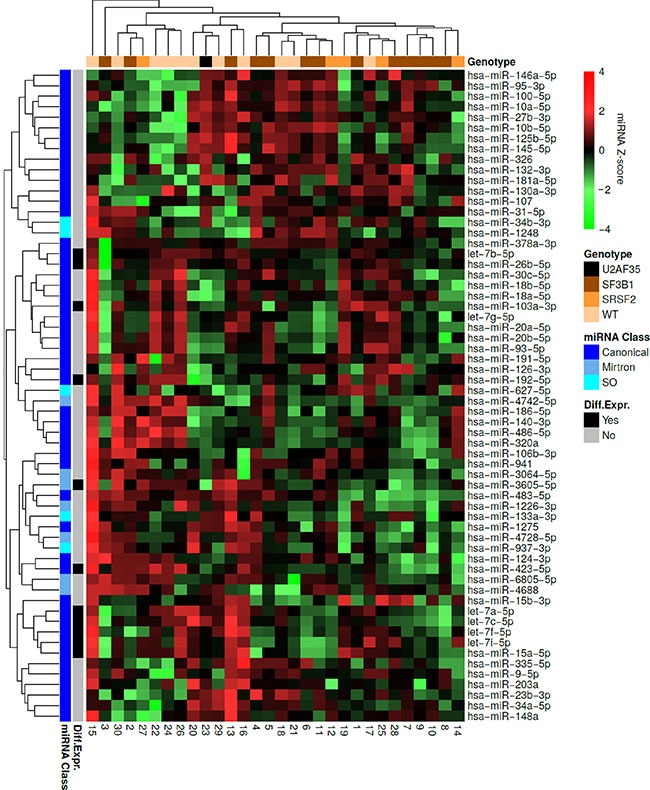
Hierarchical clustering of the MDS patient samples and miRNAs Each row represents a miRNA and each column represents a patient sample. Green indicates low expression relative to the mean expression level of the samples and red indicates high expression. The mutational status of each sample is indicated at the top. The miRNA class of each miRNA as well as differentially expressed miRNAs between wild-type MDS samples and *SF3B1* mutated samples are indicated to the left. It is observed that 14 out of 17 samples containing a spliceosome mutation within *SF3B1* or *SRSF2* clustered together. Most of the miRNAs, which were differentially expressed between wild-type MDS samples and samples containing an *SF3B1* mutation clustered together. Finally, all mirtrons except miR-4742-5p were found within the same cluster.

When performing hierarchical clustering for all samples and miRNAs we observed that 14 out of 17 samples clustering together contain a spliceosome mutation within *SF3B1* or *SRSF2* (Figure 3). This was unlikely to have happened by chance (*P* = 0.018). In addition, we observed that most mirtrons clustered together (*P* < 0.001). Sample 15 clustered separately from all other samples as it had most miRNAs overexpressed, with the exception of miR-145-5p, miR-146a-5p (located on chromosome 5q), miR-125b-5p, miR-10a-5p, miR-10b-5p, and miR-27b-3p. This patient did not carry a spliceosome mutation or a mutation in *DNMT3A*, and was the youngest of all the patients in this cohort (Supplementary Table S1).

We also performed hierarchical clustering for each class of miRNA separately. Among the canonical miRNAs, we observed that 8 out of 12 samples clustering together contain an *SF3B1* mutation in (Supplementary Figure S3). This was unlikely to have happened by chance (*P* = 0.04). When analyzing the mirtrons we observed that 11 out of 12 samples clustering together contain a spliceosome mutation (Supplementary Figure S4A). This was unlikely to have happened by chance (*P* = 0.01). No obvious clustering of spliceosome mutated samples could be observed when analyzing SO miRNAs (Supplementary Figure S4B).

**Figure 4:**
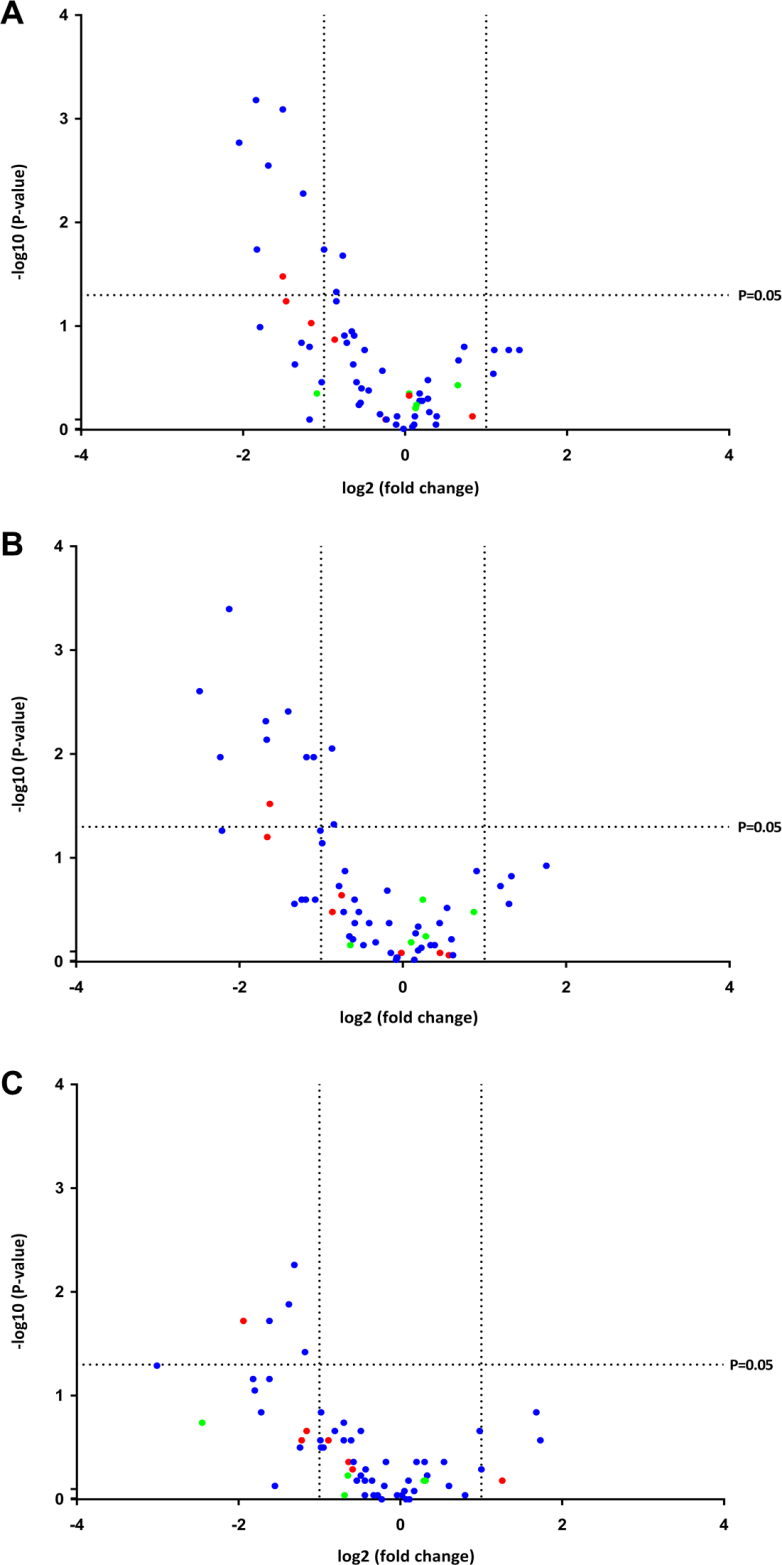
Volcano plots of the *P*-values and fold changes in expression of the miRNAs Each miRNA is represented by a colored dot indicating its class. Blue represents canonical miRNA, red represents mirtron, and green represents SO miRNA. (**A**) Spliceosome mutated samples versus wild-type samples. It can be observed that all differentially expressed miRNAs were downregulated in the spliceosome mutated samples. (**B**) *SF3B1* mutated samples versus wild-type samples. It can be observed that all differentially expressed miRNAs were downregulated in the samples containing an *SF3B1* mutation. (**C**) *SRSF2* mutated samples versus wild-type samples. It can be observed that all differentially expressed miRNAs were downregulated in the samples containing an *SRSF2* mutation.

Overall, canonical miRNAs were downregulated in spliceosome mutated samples versus wild-type MDS samples (*P* = 0.002), and there was a tendency for mirtrons to be downregulated as well (*P* = 0.11), while this was not the case for SO miRNAs (*P* = 0.63) (Figure 4A). Because each spliceosome mutation may have a different impact on the expression of miRNAs, *SF3B1* mutated samples and *SRSF2* mutated samples were analyzed separately. In *SF3B1* mutated samples canonical miRNAs were downregulated (*P* = 0.004), and there was a tendency for mirtrons to be downregulated (*P* = 0.16), while SO miRNAs were not (*P* = 0.44) (Figure 4B). Finally, it was observed that canonical miRNAs were downregulated in *SRSF2* mutated samples (*P* = 0.002), while mirtrons (*P* = 0.22) and SO miRNAs (*P* = 0.31) were not (Figure 4C).

### Identification of differentially expressed miRNAs in spliceosome mutated samples

*SF3B1* was the most frequently mutated gene and we decided to mainly focus on these mutations in the subsequent analyses. Several of the studied miRNAs were differentially expressed between wild-type samples and samples with *SF3B1* mutations, and all were downregulated in the *SF3B1* mutated samples. Most of these were canonical miRNAs, with the exception of one mirtron (miR-3605-5p). Nine of the 10 canonical miRNAs were located in host genes. Furthermore, most of these miRNAs have previously been shown to have tumor suppressor properties (Table 1).

**Table 1:** Differentially expressed miRNAs between SF3B1 mutated patients and wild-type patients

MiRNA	miRNA-class	Chr.	Host Gene	Transcribed direction	LFC	Tumor Suppressor	*P*-value
**let-7c-5p**	Canonical	21	*MIR99AHG*	Same	−2.13	Yes	0.001
**let-7b-5p**	Canonical	22	*MIRLET7BHG*	Same	−2.49	Yes	0.002
**let-7f-5p** let-7f-1 let-7f-2					−1.4	Yes	0.004
	Canonical	9	*JB153432*	Same			
	Canonical	x	*HUWE1*	Same			
**let-7a-5p** let-7a-1 let-7a-2 let-7a-3					−1.67	Yes	0.005
	Canonical	9	*JB153432*	Same			
	Canonical	11	*MIRLET100*	Same			
	Canonical	22	*MIRLET7BHG*	Same			
**hsa-miR-423-5p**	Canonical	17	*NSRP1*	Same	−1.66	Yes	0.007
**hsa-miR-103a-3p** hsa-miR-103a-1 hsa-miR-103a-2					−0.87	Yes	0.009
	Canonical	5	*PANK3*	Same			
	Canonical	20	*PANK2*	Same			
**hsa-miR-26b-5p**	Canonical	2	*CTDSP1*	Same	−2.24	Yes	0.011
**let-7i-5p**	Canonical	12	No host gene	N/A	−1.09	Yes	0.011
**hsa-miR-15a-5p**	Canonical	13	*DLEU2*	Same	−1.18	Unknown	0.011
**hsa-miR-3605-5p**	Mirtron	1	*PHC2*	Same	−1.23	Unknown	0.030
**hsa-miR-192-5p**	Canonical	11	*AB429224*	Same	−0.84	Yes	0.047

Abbreviations, Chr: Chromosome, LFC: log2 fold change.

**Figure 5:**
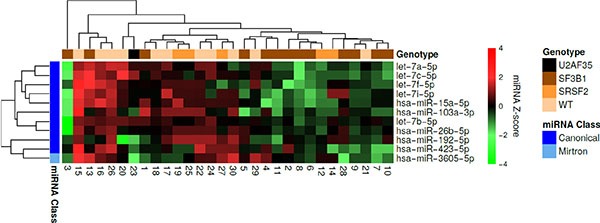
Hierarchical clustering of the MDS patient samples and differentially expressed miRNAs between wild-type samples and samples containing an *SF3B1* mutation Each row represents a miRNA and each column represents a patient sample. Green means low expression relative to the mean expression level of the samples and red means high expression. The mutational status of each sample is indicated at the top. It can be observed that the majority of the miRNAs were downregulated in the majority of the *SF3B1* mutated samples.

Hierarchical clustering was performed for the differentially expressed miRNAs, and the majority of the miRNAs were downregulated in the majority of the *SF3B1* mutated samples (Figure 5). Sample 1 and 13 were exceptions to this trend. Of note, sample 13 carried a *DNMT3A* mutation, and diverted the most from the other *SF3B1* mutated samples. Interestingly, this sample clustered together with wild-type samples, and was the only sample carrying an R625G mutation.

Only five miRNAs (let-7a-5p, let-7b-5p, let-7c-5p, miR-335-5p, and miR-4728-5p) were differentially expressed in *SRSF2* mutated compared to wild-type MDS samples. Except for one mirtron (miR-4728-5p) all of these miRNAs were intragenic canonical miRNAs transcribed in the same direction as their host genes and downregulated in the mutated samples. Three of these miRNAs (let-7a-5p, let-7b-5p, and let-7c-5p) were also significantly downregulated in *SF3B1* mutated samples relative to wild-type samples (Table 1). When considering all mutated samples together, eight miRNAs (let-7c-5p, let-7a-5p, let-7b-5p, miR-423-5p, let-7f-5p, miR-26b-5p, miR-15a-5p, and miR-3605-5p) were differentially expressed compared to wild-type samples. Except for one mirtron (miR-3605-5p), all of these miRNAs were intragenic canonical miRNAs downregulated in the mutated samples. All of these were also significantly downregulated in *SF3B1* mutated samples relative to wild-type samples, which is not surprising as most of the spliceosome mutated samples had an *SF3B1* mutation.

### Target and pathway analyses of the differentially expressed miRNAs

miRNA target predictions were performed for the differentially expressed miRNAs between wild-type samples and *SF3B1* mutated samples. The miRNA target predictions of the differentially expressed miRNAs were compared to target predictions of the remaining miRNAs. These analyses revealed many putative oncogenes among the significantly enriched targets (See Supplementary Table S2), underscoring the notion that the differentially expressed miRNAs are tumor suppressors.

Next, it was investigated which cellular processes and human diseases the significantly enriched predicted targets are involved in using the Kyoto Encyclopedia of Genes and Genomes (KEGG) pathway database. Several KEGG pathways related to MDS were found among the top significant hits (*P* < 0.001) including apoptosis, hematopoietic cell lineage, acute myeloid leukemia, JAK-STAT signaling pathway, spliceosome, MAPK signaling pathway, p53 signaling pathway, chemokine signaling pathway, and several cancers. In addition, several metabolic pathways were found among the top hits including glutathione metabolism, beta-alanine metabolism, alanine, aspartate and glutamate metabolism, glycolysis/gluconeogenesis, amino sugar and nucleotide sugar metabolism, and citrate cycle (TCA cycle) (See Supplementary Table S3).

### Host gene analyses

To investigate whether the observed downregulation of miRNAs could be a result of their respective host genes being downregulated each canonical miRNA was classified as intergenic or intragenic (Supplementary Figure S5A). Only one of the significantly downregulated miRNAs was intergenic and, overall, there was no difference in expression between intergenic and intragenic miRNAs (*P* = 0.86) (Supplementary Figure S5B). Next, the expression of four host genes *MIRLET7BHG*, *CTDSP1*, *PANK2*, and *PANK3* were measured. *MIRLET7BHG* is host gene for let-7b and *CTDSP1* for miR-26b, while *PANK2* and *PANK3* host miR-103a-2 and miR-103a-1, respectively. A weak but non-significant positive correlation between let-7b and *MIRLET7BHG* was observed (*P* = 0.08), while no correlations were observed between the expression of *CTDSP1* and miR-26b and between *PANK2* and *PANK3* and miR-103a (Supplementary Figure S6).

### Validation of the custom plate miRNA data using single TaqMan assays

Individual cDNA syntheses were performed twice for all samples and analyzed by single TaqMan assays for the following miRNAs: let-7b, miR-10b-5p, miR-26b-5p, miR-29a-5p, miR-103-3p, miR-130b-3p, miR-145-5p, and miR-146a-5p. A high reproducibility of the raw Ct-values obtained after RT-qPCR performed on individual cDNA syntheses for each miRNA was observed (Supplementary Figure S7A). Furthermore, relatively good correlations were observed between the normalized data of the single TaqMan assays and the normalized data from the 96-well custom plate (Supplementary Figure S7B). Finally, all of these miRNAs were downregulated in patients with *SF3B1* mutations relative to wild-type patients as expected, with the exception of miR-10b-5p, which was also upregulated in the previous analysis (Supplementary Figure S8).

## DISCUSSION

A high frequency of spliceosome mutations are observed in MDS [8]. Therefore, it is likely that abnormal RNA splicing plays an important role in MDS pathogenesis. Spliceosome mutations in MDS are generally gain-of-function in nature, but little is known about their potential effects on the transcriptome [19]. It has, however, been shown that the vast majority of differentially expressed genes in *SF3B1* mutated samples compared to wild-type samples are downregulated [20, 21]. This is likely due to the use of cryptic 3′ splice sites leading to retention of an intronic sequence containing *in frame* stop codons, which, in turn, may lead to nonsense-mediated mRNA decay (NMD) [20]. Alternatively, it may be due to key regulatory genes (e.g. encoding transcription factors or epigenetic modifiers) being aberrantly spliced. Splicing abnormalities of the epigenetic modifiers *ASXL1* and *EZH1*, the transcription factor *RUNX3*, as well as the RAS pathway gene *CBL* have been observed in cases with *SF3B1* mutations [21, 22]. Likewise, a splicing abnormality of *RUNX1*, a recurrently mutated transcription factor in MDS, has been observed in bone marrow cells from two cases with *SRSF2* mutations, and aberrant splicing of the epigenetic modifier *TET2* has been associated with mutations in *U2AF35* [4]. In addition, *SF3B1* mutations often coexist with mutations in *DNMT3A* [8], and for this reason it can be difficult to decipher the *in vivo* effects of each individual mutation.

Our study is the first to analyze the impact of spliceosome mutations on the expression of miRNAs. We decided to focus on MDS as spliceosome mutations are common in this disease and on miRNAs since they play important roles in both solid and hematologic cancers [9, 23]. We studied a large panel of miRNAs, including intragenic and intergenic canonical miRNAs previously implicated in MDS, as well as mirtrons, tailed mirtrons, and SO miRNAs. To accurately quantify the expression of the miRNAs we used the GeNorm and NormFinder algorithms to identify stably expressed miRNA genes to normalize the RT-qPCR data. These two algorithms are inherently different, but can be expected to give similar results [24]. Indeed, we observed a striking similarity between the data obtained by each algorithm. Thus, the miRNA genes we identified to be stably expressed in MDS may be useful for normalization of RT-qPCR data in future miRNA studies. On the other hand, the commonly used references, *SNORD44* and *SNORD47*, were not among the most stable genes in either of the analyses.

Overall, our results show that miRNA expression is affected by spliceosome mutations in MDS. In line with the observations by Visconte and colleagues [21], we observed a strong association between spliceosome mutations and miRNA downregulation. In fact, we found none of the miRNAs to be significantly upregulated in *SF3B1* or *SRSF2* mutated samples compared to wild-type samples. Because mutations within *SF3B1* coexist with mutations in *DNMT3A* the observed miRNA downregulation could, in theory, be caused by altered methylation patterns of regulatory regions associated with the miRNAs and/or their host-genes. Thus, we tested all samples for mutations within *DNMT3A*. However, only three of the *SF3B1* mutated samples carried a *DNMT3A* mutation, making it highly unlikely that *DNMT3A* mutations are responsible for the observed downregulation of miRNAs in the spliceosome mutated samples. Furthermore, two of the samples (patient 3 and 13), which carried both an *SF3B1* mutation and a *DNMT3A* mutation, clustered together with wild-type samples. This cluster analysis also indicates that spliceosome mutations have an overall effect on the transcription of miRNAs, as most spliceosome mutated samples clustered together. Finally, it should be noted that the spliceosome is a very complex macromolecule consisting of numerous proteins and snRNAs [25], and it cannot be ruled out that wild-type samples carry a mutation in other spliceosome genes apart from the most commonly mutated studied here.

When analyzing each miRNA class separately it was observed that none of the SO miRNAs were significantly downregulated. This was expected since the Drosha/DGCR8 complex competes with the spliceosome machinery for processing of the precursor RNA molecule. Thus, it is likely that a mutated spliceosome is less competitive, leading to increased production of the miRNA and less production of the mRNA. However, as few SO miRNAs were expressed in the samples we were unable to make firm conclusions regarding the influence of spliceosome mutations on this group of miRNAs. On the other hand, it was expected that less efficient splicing would lead to decreased expression of mirtrons, which are directly dependent on the spliceosome for processing of the precursors. Indeed, it could be observed that most mirtrons were downregulated in spliceosome mutated samples compared to wild-type samples. However, we were surprised to observe that the spliceosome mutations had most profound effect on canonical miRNAs.

Only two of the differentially expressed miRNAs in our study are directly processed by the spliceosome (miR-3605-5p and miR-4728-5p), and we found an intergenic miRNA (let-7i-5p) to be significantly downregulated in the *SF3B1* mutated samples. These observations point towards indirect mechanisms mainly being responsible for the aberrant expression of miRNAs in spliceosome mutated MDS. This could potentially be due to aberrant splicing of genes encoding transcription factors or epigenetic modifiers, which, in turn, may have an effect on the expression of miRNA host genes as well as miRNA genes having their own promotors. A weak but non-significant positive correlation between let7b and *MIRLET7BHG* (*P* = 0.08) was found, while no correlations were observed between the expression of *CTDSP1* and miR-26b and between *PANK2* and *PANK3* and miR-103a. It was, however, expected that correlations between *PANK2* and *PANK3* and miR-103a would be weaker as miR-103a has two genes, miR-103a-1 and miR-103a-2, located in *PANK3* and *PANK2*, respectively. Furthermore, both genes have antisense transcripts, which may also influence the expression of the mature miRNA. Nevertheless, strong correlations between miRNAs and their host genes would not be expected as their half-lives are likely to be dissimilar and most host genes have several isoforms which may be differentially expressed.

Interestingly, we found several tumor suppressor miRNAs to be downregulated in patient samples with *SF3B1* and *SRSF2* mutations. Among these were several miRNAs belonging to the let-7 family, which are well known for their tumor suppressor activities [26, 27], and their essential role in stem cell differentiation and hematopoiesis [28]. In MDS let-7a has been shown to downregulate oncogenic KRAS and to be under-expressed in patients with intermediate or high-risk karyotype [29]. The miR-26 family is also involved in hematopoiesis [9], and miR-423-5p and miR-103a have previously been shown to be downregulated in MDS and to have tumor suppressor properties [30, 31]. Interestingly, a recent study highlighted that several genes involved in early hematopoiesis are aberrantly spliced in a model of mutant *SRSF2* [32], including *MEIS1* [33]. Thus our data suggest that the ineffective hematopoiesis observed in MDS not only results from aberrant splicing of protein encoding genes, but also from downregulation of miRNAs.

When comparing the predicted targets of the differentially expressed miRNAs with the remaining miRNAs many putative oncogenes were among the significantly enriched targets. These included *BACH1*, a transcription factor, which is upregulated in several cancers and known to be regulated by the let-7 family [34], *HMGA1*, which is involved in cell differentiation and apoptosis and upregulated in several cancers [35], *SLC4A4*, which is upregulated in chronic myeloid leukemia stem cells [36], *ZBTB5*, which function as an oncogene by repressing the cell cycle arrest gene *p21* [37], and *ACSL6*, which has been shown to be part of a gene fusion in MDS and polycythemia vera [38, 39]. Overall, the significantly enriched predicted targets were found to be involved in apoptosis, hematopoiesis, acute myeloid leukemia, the JAK-STAT-, MAPK-, p53-, and chemokine signaling pathways, the spliceosome, and several cancers. In addition, several metabolic pathways were among the most significant pathways, which corresponds well with the recent finding that let-7a plays a prominent role in regulating energy metabolism in cancer cells [40].

In conclusion, our study is the first to provide evidence that spliceosome mutations play an important role in MDS pathophysiology by affecting the expression of tumor suppressor miRNA genes involved in the development and progression of MDS.

## MATERIALS AND METHODS

### Patient samples

Ten mL of bone marrow (BM) were aspirated from the posterior iliac crest of 34 MDS patients at the time of diagnosis and three subjects with suspected MDS due to peripheral cytopenia, but without BM dysplasia or chromosomal abnormalities. The diagnoses of MDS were based on morphology supplemented by cytogenetics, and divided into subgroups according to WHO 2008 criteria.

Clinical and cytogenetic data of MDS patients can be found in Supplementary Table S1.

The study was approved by the ethical committee for the Capital Region of Denmark (H-D-2009-003) and the Danish Data Protection Agency (30–1419) and conducted in accordance with the tenants of Helsinki.

### Extraction of DNA and miRNA

DNA was purified from the mononuclear fraction of BM samples using the Gentra Puregene Cell Kit (Qiagen) according to the manufacturer's instructions. miRNA was purified from Formalin-Fixed Paraffin Embedded (FFPE) whole BM biopsies using the Recover All Total Nucleic Acid Isolation Kit for FFPE (Ambion) according to the manufacturer's instructions.

### Mutation detection

HRM assays, covering mutations within *SF3B1* (3 hotspots), *SRSF2* (1 hotspot), *U2AF35* (2 hotspots) and *DNMT3A* (3 hotspots), were designed and optimized. DGGE assays, covering mutations within *SF3B1* (3 hotspots) were designed and optimized. All PCR primer sequences are shown in Supplementary Table S4. The nature of the mutations detected in the samples was confirmed using traditional Sanger sequencing of the PCR products. Additional information can be found in the Supplementary Files.

### TaqMan miRNA 96-well custom plate design

The miRNA TaqMan assays used in this study were pre-spotted on a 96-well plate. In total, 67 canonical miRNAs, 11 mirtrons, and 11 SO miRNAs were included, together with four potential noncoding RNA reference genes. A background signal was detected for 17 assays, which were excluded from further analyses, yielding a total of 56 canonical miRNAs, nine mirtrons, 11 SO miRNAs, and three potential noncoding RNA reference genes. The miRNAs were selected following a literature search to ensure that the majority has been implicated in MDS previously. Details regarding each miRNA assay can be found in Supplementary Table S5.

### RT-qPCR

One-hundred ng of RNA from each patient sample were reverse transcribed with the TaqMan^®^ MicroRNA Reverse Transcription Kit (Life Technologies) according to manufacturer's instructions with a customized primer pool for all miRNA targets. The reactions were performed on a Veriti^®^ 96-well Thermal Cycler (Applied Biosystems) with the following conditions: one cycle of 16°C for 30 min, 42°C for 30 min, and 85°C for 5 min. Prior to qPCR the cDNA samples were pre-amplified using TaqMan^®^ PreAmp Master Mix Kit (Applied Biosystems) according to the manufacturer's instructions. The pre-amplification was performed on a Veriti^®^ 96-well Thermal Cycler (Applied Biosystems) with the following conditions: one cycle of 95°C for 10 min, 55°C for 2 min, 72°C for 2 min, 12 cycles of 95°C for 15 sec and 60°C for 4 min, and one cycle of 99.9°C for 10 min. Subsequently, the samples were diluted 1:10 in DNAse/RNAse free water. cDNA synthesis and pre-amplification were also performed on a no enzyme control (NEC) sample and a no template control (NTC). The qPCRs were performed using the TaqMan^®^ Universal Master Mix II, no UNG Kit (Life Technologies) in a 96-well format, as described above, according to the manufacturer's instructions on a StepOnePlus^®^ Real-Time PCR System (Life Technologies). The following negative controls were included; the NEC and NTC from the cDNA synthesis, NTC from the pre-amplification and NTC at the qPCR level.

### Host gene analyses

Single TaqMan assays were used for the host gene analysis. In total, four host genes and two reference genes were included in the study. In the RT-qPCR step ten ng of RNA from each patient sample were reverse transcribed with the TaqMan^®^ MicroRNA Reverse Transcription Kit (Life Technologies) according to the manufacturer's instructions with a primer pool for all target genes. The reactions were performed on a Veriti^®^ 96-well Thermal Cycler (Applied Biosystems) as described above. Prior to qPCR the cDNA samples were pre-amplified using TaqMan^®^ PreAmp Master Mix Kit (Applied Biosystems) according to the manufacturer's instructions. The pre-amplification was performed as described above using 10 instead of 12 cycles of 95°C for 15 sec and 60°C for 4 min. Subsequently, the samples were diluted 1:5 in DNAse/RNAse free water. The qPCRs were performed using the TaqMan^®^ Universal Master Mix II, no UNG Kit (Life Technologies), according to the manufacturer's instructions on a StepOnePlus^®^ Real-Time PCR System (Life Technologies). The same negative controls as described above were included in these experiments.

### Validation of the custom plate miRNA data using single TaqMan assays

RT-qPCR was performed using single TaqMan assays (Life Technologies) for the following miRNAs; let-7b, miR-10b-5p, miR-26b-5p, miR-29a-5p, miR-103-3p, miR-130b-3p, miR-145-5p, and miR-146a-5p. cDNA synthesis, pre-amplification, and qPCR were performed in duplicates for each patient sample. Ten ng of RNA were reverse transcribed using the TaqMan^®^ MicroRNA Reverse Transcription Kit according to manufacturer's instructions (Life Technologies). For pre-amplification all eight reverse transcription primers were pooled according to manufacturer's instructions. The RT reaction was as described above. The pre-amplification was performed as described above using 10 instead of 12 cycles of 95°C for 15 sec and 60°C for 4 min. Subsequently, the samples were diluted 1:5 in DNAse/RNAse free water. The same negative controls as described above were included in these experiments.

### Data and statistical analyses

Raw cycle threshold (Ct) values for miRNAs were extracted from the StepOnePlus^®^ Real-Time PCR system (Applied Biosystems). Samples with an overall poor quality (geometric mean (Ct_ref genes_) > 35) were excluded from further analyses. Therefore, it was not due to analysis failure when a sample did not amplify for a miRNA of interest, but rather it meant that the miRNA was not expressed in that sample. Accordingly, samples not amplifying for a given miRNA were given a Ct value of 40. Stably expressed reference genes were determined by importing Ct values transformed into a linear scale expression quantities, and raw Ct values into the NormFinder [17] and GeNorm algorithms [18], respectively. Data for 29 miRNAs and three commonly used reference genes (*SNORD44*, *SNORD47*, and *RNU6B*), which were expressed in all samples, were included in these analyses. For the NormFinder analysis three biological groups (wild-type controls, wild-type MDS, and spliceosome mutated MDS) were specified. miRNA expression data were analyzed with the qBase+ software, version 3.0 (Biogazelle) using the 2^−ΔΔCt^ method [41], where ΔΔCt = (Ct_target_-Ct_geometric mean,ref genes_)_MDS sample_-(Ct_target_-Ct_geometric mean,ref genes_)_mean of control samples_. One-sided Fisher's exact tests were used to evaluate if specific clusters were enriched for a given type of sample of miRNA. Differences in miRNA expression between spliceosome mutated samples and wild-type samples were evaluated with a Mann-Whitney *U* test. Overall differences in miRNA expression between spliceosome mutated samples and wild-type samples were evaluated with a Wilcoxon signed-rank test. For the cluster analyses miRNAs were excluded from the analyses when more than 1/3 of the samples did not amplify. In total, less than five percent of the data points in the unsupervised cluster analysis were derived from samples which did not amplify. The Z-transformed miRNA expression levels were subjected to complete-linkage hierarchical clustering using the Pearson distance metric. miRNA target predictions were retrieved from TargetScan [42], PicTar [43], miRanda [44], and StarMirDB [45] and integrated to improve the precision of the predictions using the same strategy as in STRING [46]. The top 0.1% of the integrated predictions (∼100 targets per miRNA) was used to infer enriched targets and associated KEGG pathway database terms (http://www.genome.jp/kegg/pathway.html) of the differentially expressed miRNA between samples with *SF3B1* mutations compared to wild-type samples. Enrichments were evaluated using the Fisher's exact test and the Benjamini and Hochberg correction for multiple testing.

For the host gene analysis, the RT-qPCR data were analyzed as described above, using the mean Ct value of two reference genes (*UBC* and *JUNB*). Goodness-of-fit linear regression was used to evaluate possible relations between expression of the host genes and the miRNAs using Prism 6 (GraphPad software, San Diego, CA, USA), which employs an *F* test to evaluate if the slope is significantly different from zero.

For the validation analysis, the RT-qPCR data were analyzed as described above, using the mean Ct value of the two replicates for each miRNA. However, samples with a standard deviation above two for any of the reference miRNA genes were excluded from the analysis of differentially expressed miRNAs (this applied to sample 18, 23, 29, and 31). Goodness-of-fit linear regression was used to evaluate possible relations between expression of the host genes and the miRNAs using Prism 6 (GraphPad).

Any differences were considered to be statistically significant when the *P*-value was < 0.05.

## SUPPLEMENTARY MATERIALS FIGURES AND TABLES


