# Supplementary material for: Tumor suppressor microRNAs are downregulated in myelodysplastic syndrome with spliceosome mutations
Source: Oncotarget. 2016 Feb 2;7(9):9951–63. doi: 10.18632/oncotarget.7127 (PMC4891095; doi:10.18632/oncotarget.7127)
Supplement: Supplementary file 1 [file oncotarget-07-09951-s001.pdf]

Tumor suppressor microRNAs are downregulated in myelodysplastic syndrome with spliceosome mutations

Supplementary Materials

MATERIALS AND METHODS

Mutation detection

For the high-resolution melting (HRM) analyses the LightCycler® 480 instrument II (Roche) was used for real-time PCR and HRM analyses. The High Resolution Melting Master Kit (Roche) was used at a final concentration of 1X, with 2.5 mM $MgCl_2$, 200 nM of each primer, and 1 µL of genomic DNA (12.5 ng/µL) resulting in a total volume of 20 µL. The cycling protocols started with one cycle of 95°C for 10 min, followed by 45 cycles of 95°C for 10 s, 60°C for 20 s, and 72°C for 20 s, one cycle of 95°C for 1 min, one cycle of 40°C for 1 min, and a melting step from 60°C to 95°C with 20 acquisitions per °C. However, the *SRSF2* assay was performed using an annealing temperature of 65°C instead of 60°C for 20 s. Mutations were confirmed by Sanger sequencing. This was done by performing a second PCR with the same primers as for the HRM analyses, but in these PCRs the forward primers were M13-tagged

(5′-CGTTGTAAAACGACGGCCAGT) to create longer PCR products, which could be successfully sequenced. The HotStarTaq Master Mix Kit (Qiagen) was used at a final PCR buffer concentration of 1X, with 1.5 mM $MgCl_2$, 667 nM of each primer, 3.33 units/reaction HotStarTaq DNA Polymerase, and 2 µL of PCR product, resulting in a total volume of 30 µL. The cycling protocols started with one cycle of 95°C for 15 min, followed by 30 cycles of 95°C for 10 s, 60°C for 20 s, and 72°C for 20 s. However, the *SRSF2* assay was performed using an annealing temperature of 65°C instead of 60°C for 20 s. These PCR products were sequenced in the forward direction by the commercial services of Eurofins using an M13 primer identical to the M13-tag used.

For the denaturing gradient gel electrophoresis (DGGE) analyses the Veriti® 96-well Thermal Cycler (Applied Biosystems) was used for PCR amplification. The HotStarTaq Master Mix Kit (Qiagen) was used at a final PCR buffer concentration of 1X, with 1.5 mM $MgCl_2$, 667 nM of each primer, 3.33 units/reaction HotStarTaq DNA Polymerase, and 1 µL of genomic DNA (12.5 ng/ µL) resulting in a total volume of 15 µL. The same PCR cycling protocol was used as for the HRM assays. Twelve ml of the GC-clamped PCR products were loaded onto a denaturing gradient gel containing a gradient of 6% to 12% polyacrylamide gel and of urea and formamide from 10% to 70%, which increased towards the positive electrode. The gels were run at 170 V for 4.5–5 hours in 1X TAE buffer kept at a constant temperature of 57°C. After electrophoresis, the gel was stained in TAE buffer containing ethidium bromide for 5 min and photographed by ultraviolet (UV)-transillumination (Vilbur Lourmat). The nature of the mutations detected in the samples using DGGE was also confirmed using traditional Sanger sequencing of the PCR products. The heteroduplex bands were cut out of the gels and dissolved in 100 µL DNAse/ RNAse free water by vortexing. A second PCR with the same primers as for the DGGE analyses, but without the GC-clamps, was performed. The reaction- and cycling conditions were as described above for the second PCR following the HRM analyses. These PCR products were sequenced in both forward and reverse directions by the commercial services of Eurofins using the same primers as for the second PCR.

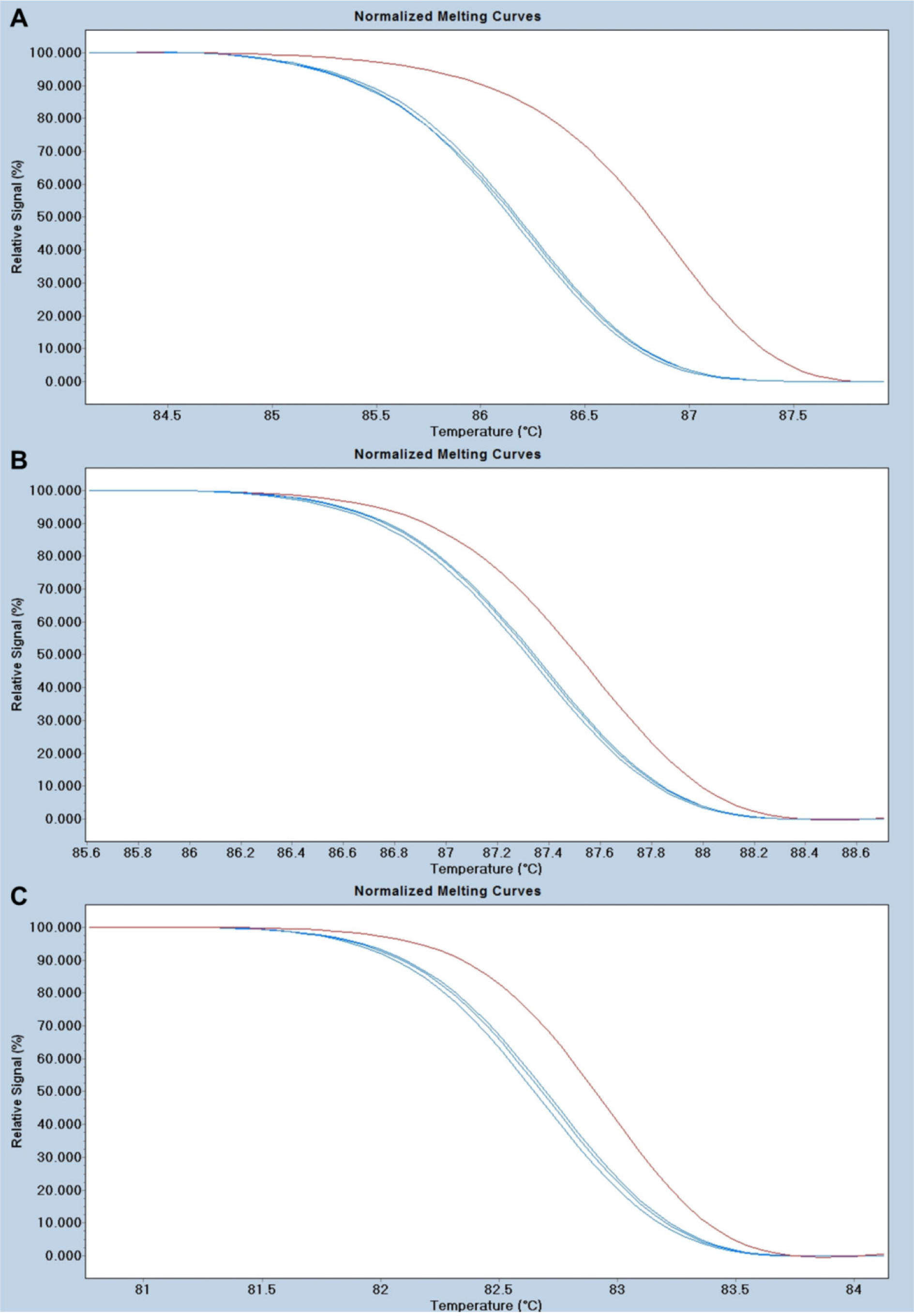

Supplementary Figure S1: High-resolution melting assays for the detection of *DNMT3A* mutations. Representative data for each of the hotspots are shown. (**A**) *DNMT3A* hotspot containing the exon 20 mutation detected in sample 11 (represented by the red curve). (**B**) *DNMT3A* hotspot containing the exon 21 mutation detected in sample 3 (represented by the red curve). (**C**) *DNMT3A* hotspot containing the exon 22 mutation detected in sample 12 (represented by the red curve).

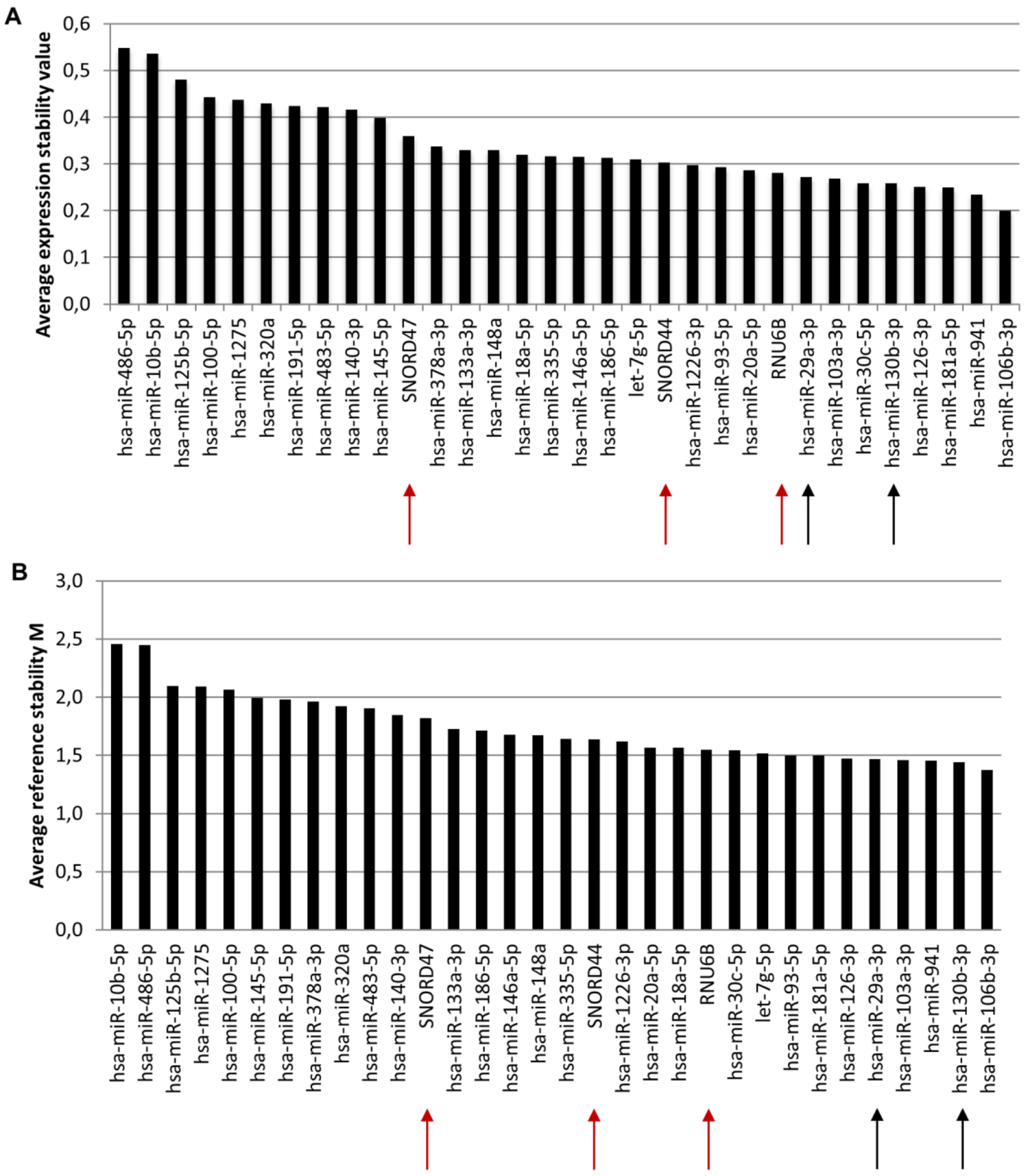

Supplementary Figure S2: Identification of stably expressed reference miRNA genes. Data for 29 miRNAs and three commonly used reference genes (*SNORD44, SNORD47, RNU6B*), were included in the analyses. (**A**) The NormFinder analysis showed that the best combination of any two reference miRNA genes were miR-29a-3p and miR-130b-3p (indicated by black arrows), while the single most stable gene was miR-106b-3p. The commonly used reference genes are indicated by red arrows. (**B**) The GeNorm analysis showed that the single most stable gene was miR-106b-3p. It can be observed that miR-29a-3p and miR-130b-3p (indicated by black arrows) were among the most stable genes in the GeNorm analysis. The commonly used reference genes are indicated by red arrows.

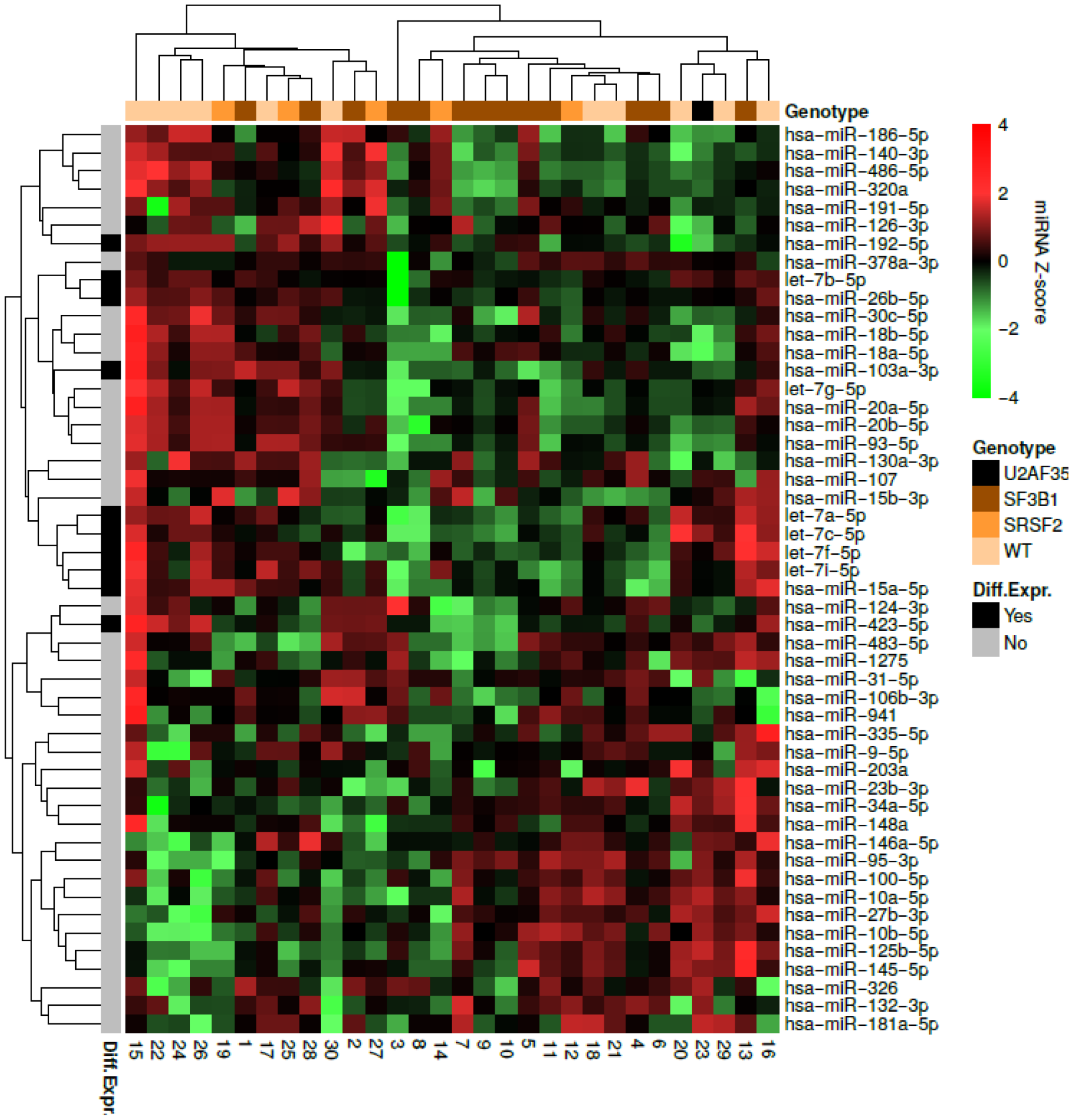

Supplementary Figure S3: Hierarchical clustering of the MDS patient samples and canonical miRNAs. Each row represents a miRNA and each column represents a patient sample. Green indicates low expression relative to the mean expression level of the samples and red indicates high expression. The mutational status of each sample is indicated at the top. It can be observed that 10 out of 12 samples clustering together contain a spliceosome mutation within *SF3B1* or *SRSF2*.

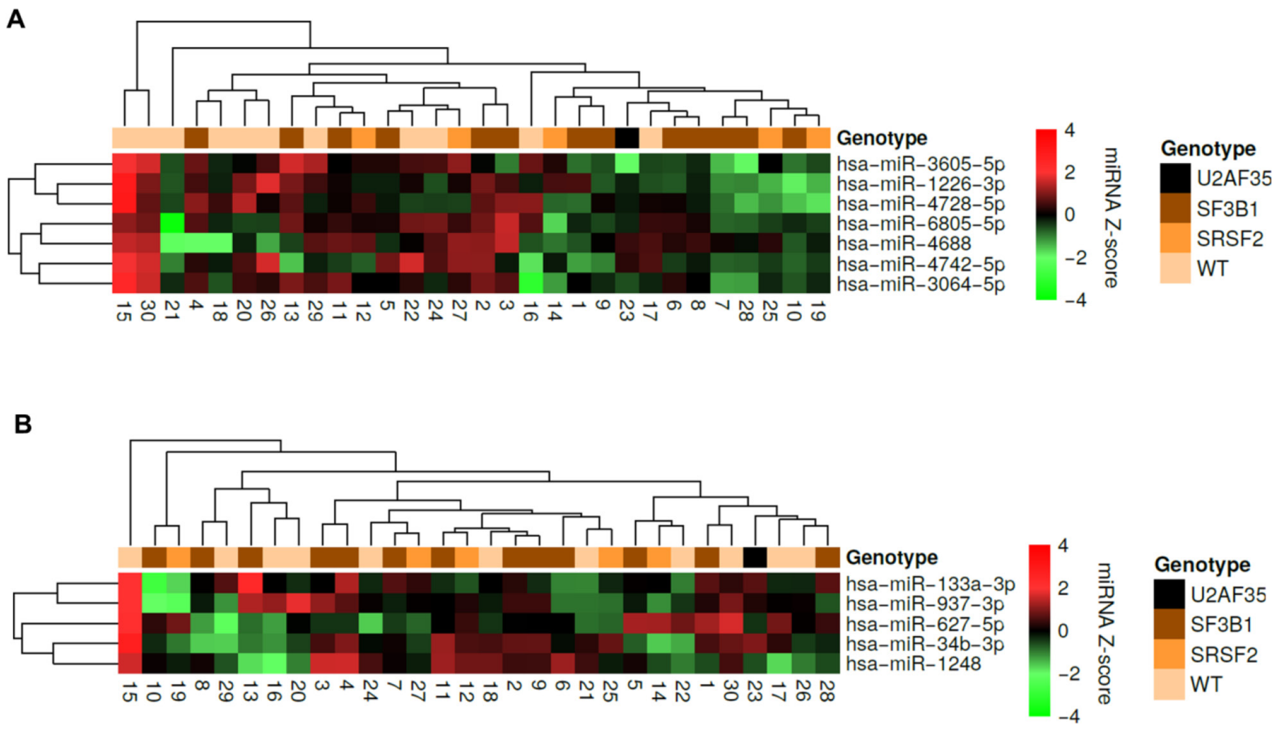

Supplementary Figure S4: Hierarchical clustering of the MDS patient samples and mirtrons and SO miRNAs. Each row represents a miRNA and each column represents a patient sample. Green indicates low expression relative to the mean expression level of the samples and red indicates high expression. The mutational status of each sample is indicated at the top. (**A**) The cluster analysis contained only mirtrons. It can be observed that 11 out of 12 samples clustering together contain a spliceosome mutation. (**B**) The cluster analysis contained only SO miRNAs. No obvious clustering of spliceosome mutated samples could be observed.

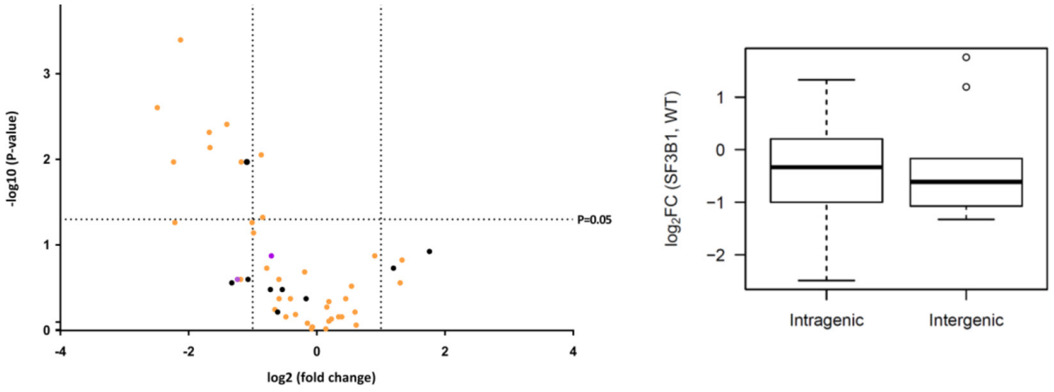

Supplementary Figure S5: Comparison of intergenic and intragenic canonical miRNAs in *SF3B1* mutated samples versus wild-type. (**A**) Volcano plot of the *P*-values and fold changes in expression. Each miRNA is represented by a colored dot indicating its class. Orange indicates that the miRNA is located within a host gene (intragenic), black indicate that the miRNA is intergenic, and purple indicate that the miRNA has both intergenic and intragenic transcripts. (**B**) Box plots of the same data shown in (A), except the two miRNAs with both intergenic and intragenic transcripts. Overall, there was no statistically significant difference in the expression between intergenic and intragenic miRNAs ($P = 0.86$).

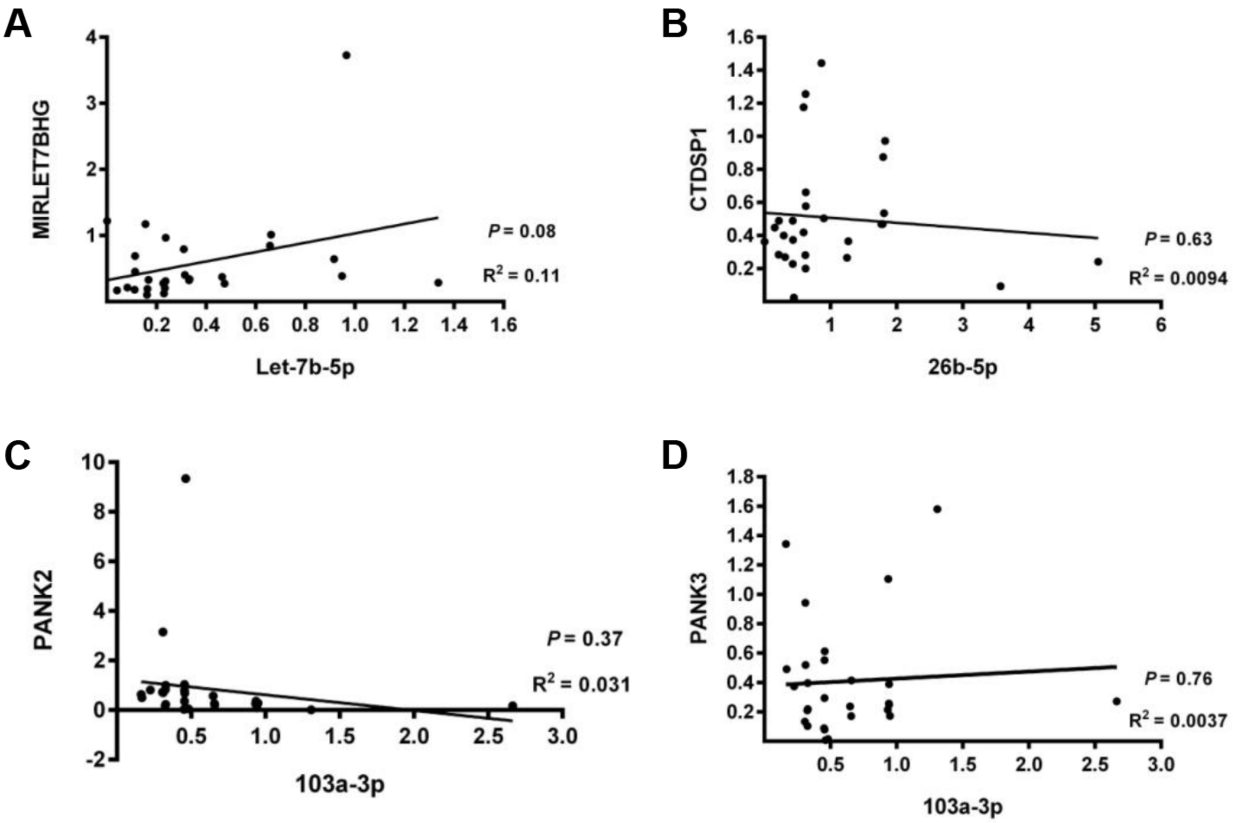

Supplementary Figure S6: Expression analyses of miRNAs relative to the expression their respective host genes. Normalized data from the 96-well custom plate were plotted against the normalized data from single TaqMan assays for the host genes. (**A**) MIRLET7BHG versus Let-7b-5p. (**B**) CTDSP1 versus miR-26b-5p. (**C**) PANK2 versus miR-103a-3p. (**D**) PANK3 versus miR-103a- 3p.

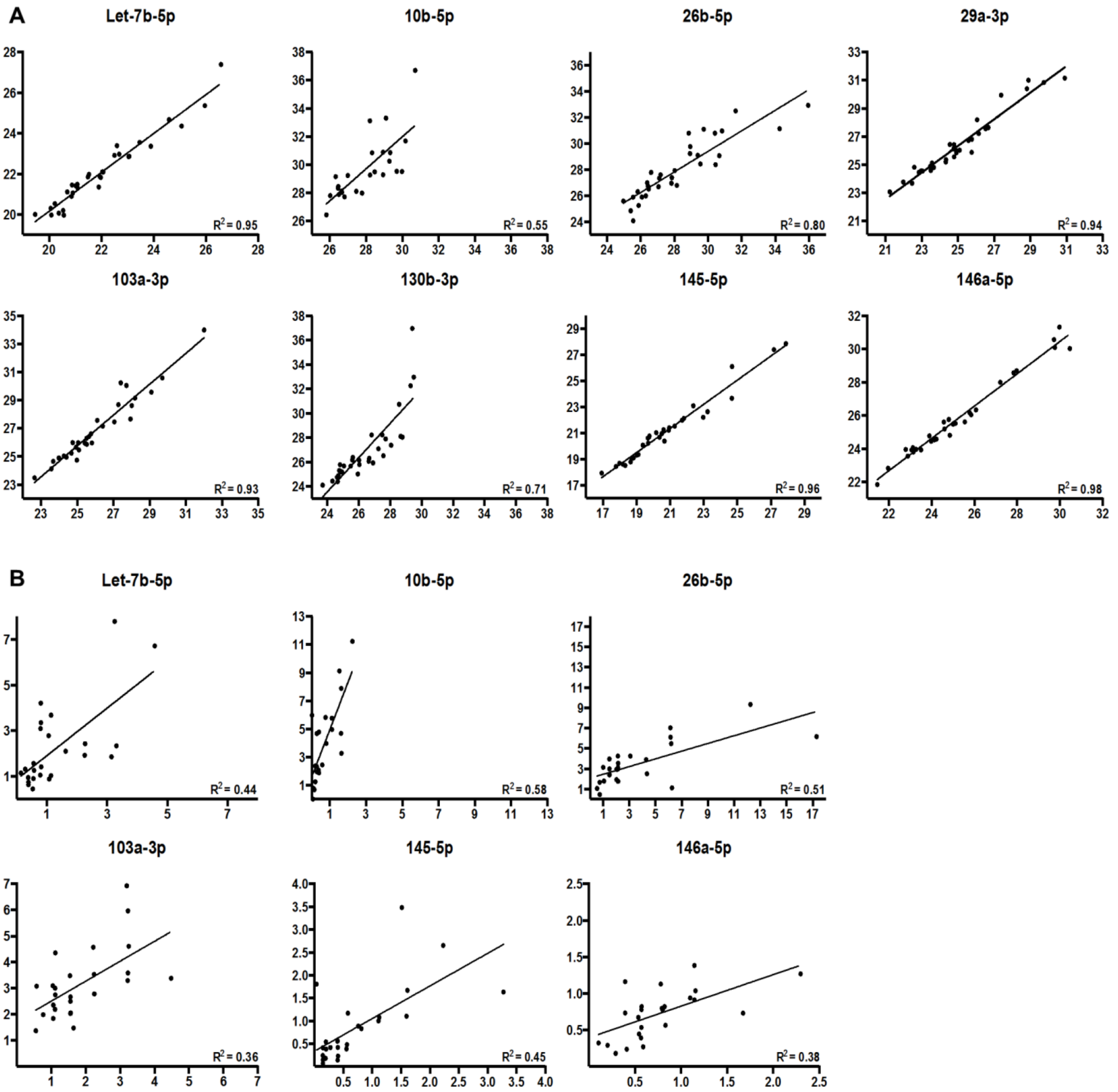

Supplementary Figure S7: Validation of the custom plate miRNA data using single TaqMan assays. (**A**) Individual cDNA syntheses were performed twice for all samples, and the reproducibility evaluated. Raw Ct-values for the two individual cDNA syntheses were plotted against each other. (**B**) Normalized data of the single TaqMan assays were plotted against the normalized data from the 96-well custom plate. Ct values from the TaqMan assays are on the x-axis and Ct values from the 96-well custom plate are on the y-axis.

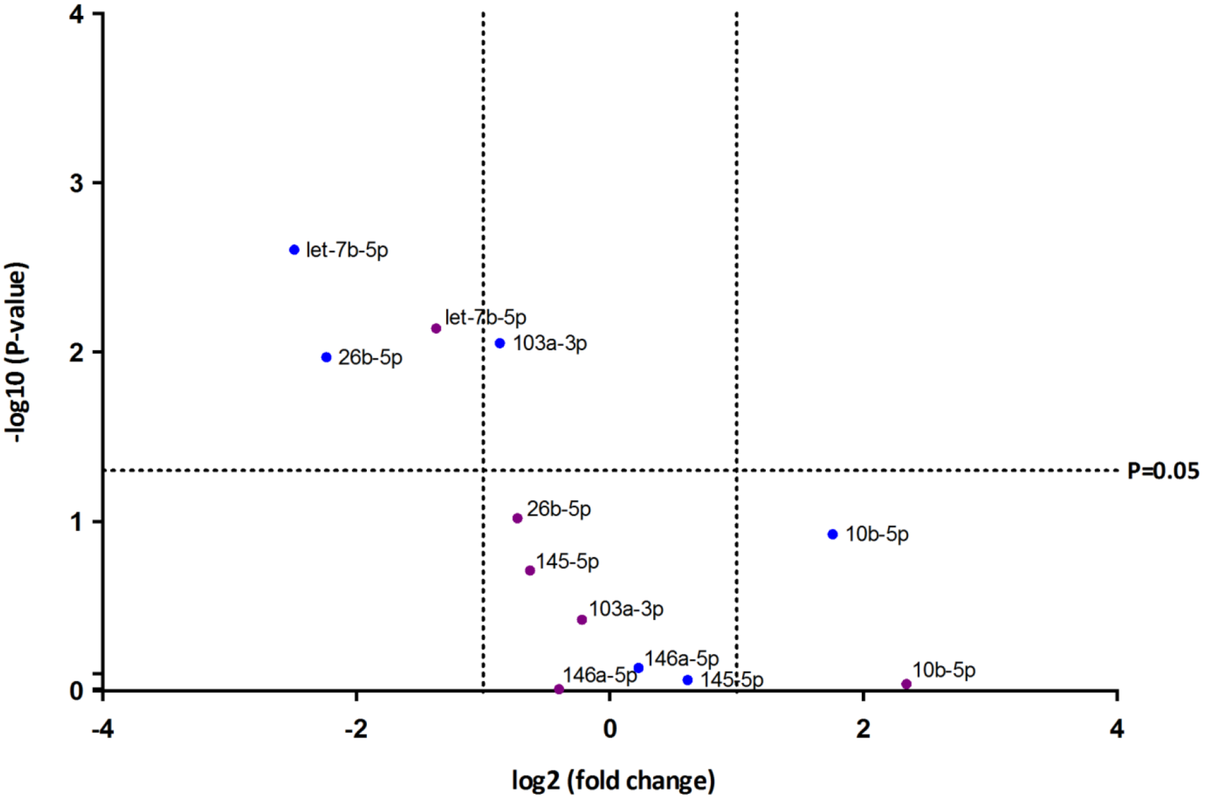

Supplementary Figure S8: Volcano plots of the *P*-values and fold change in expression between wild-type samples and samples containing an *SF3B1* mutation for each of the miRNAs studied by single TaqMan assays compared to the data from the 96-well custom plate. Each miRNA is represented by a colored dot. Blue indicates results from the 96-well custom plate, and purple indicates results from the single TaqMan assays.

Supplementary Table S1: Mutational status and clinical characteristics of the studied MDS patients

Patient no.	Gender	Age	Spliceosome mutations	*DNMT3a* mutations	WHO subtype	IPSS score	IPSS group	Cytogenetics
1	Male	67	*SF3B1* K700E	WT	RARS	0	Low	46, XY [25]
2	Male	63	*SF3B1* K700E	WT	RARS	0	Low	46, XY [25]
3	Female	74	*SF3B1* K666R	Exon 21	RARS	0	Low	46, XX [26]
4	Female	84	*SF3B1* K700E	WT	RARS	0	Low	46, XX [25]
5	Male	92	*SF3B1* K666R	WT	RARS	0	Low	46, XY [25]
6	Female	80	*SF3B1* K700E	WT	RARS	0.5	Int-1	46, XX [26]
7	Female	69	*SF3B1* K700E	WT	RARS	0	Low	46, XX [27]
8	Female	79	*SF3B1* K666T	WT	RARS	0	Low	46, XX [26]
9	Male	79	*SF3B1* H662Q	WT	RARS	0.5	Int-1	46, XY, inv (9) (p22q22) [28]
10	Male	73	*SF3B1* N626D	WT	RARS	0	Low	46, XY [27]
11	Female	85	*SF3B1* K700E	Exon 20	RARS	0.5	Int-1	47, XX, +8 [22]/46, XX [4]
12	Male	84	*SRSF2* P95L	Exon 22	RARS	0	Low	No data
13	Male	76	SF3B1 R625G	Exon 22	RA	0	Low	45, X, −Y [3]/46, XY [22]
14	Male	75	*SRSF2* P95L	WT	RA	0	Low	No data
15	Female	52	WT	WT	RA	0	Low	46, XX [27]
16	Female	67	WT	WT	Del 5q	0	Low	46, XX, del(5) (q15q33) or (q22q35) [16]/46, XX [8]
17	Female	82	WT	WT	RA	0	Low	46, XX [24]
18	Male	77	WT	WT	RA	0	Low	46, XY [25]
19	Female	71	*SRSF2* P95L	WT	RA	0.5	Int-1	46, XX [25]
20	Male	90	WT	WT	RA	0	Low	46, XY [25]
21	Female	60	WT	WT	RA	0	Low	46, XX [25]
22	Female	65	WT	WT	RA	0	Low	46, XX [25]
23	Male	88	*U2AF35* Q157R	WT	RA	0	Low	46, XY [25]
24	Male	69	WT	WT	RA	0.5	Int-1	No data
25	Female	73	*SRSF2* P95L	WT	RA	0	Low	46, XX [25]
26	Female	81	WT	WT	RA	1	Int-1	46, XX, del (11) (q?) [5]/46, XX [20]
27	Male	74	*SRSF2* P95H	WT	RA	0.5	Int-1	46, XY [26]
28	Female	74	*SF3B1* E622D	WT	RA	0	Low	46, XX [26]
29	Male	92	WT	WT	RA	0	Low	46, XY, [27]
30	Male	57	WT	WT	RA	0	Low	46, XY [27]
31	Female	63	*SF3B1* K700E	WT	RARS	0.5	Int-1	47, XX, −8 [3]/46, XX [25]
32	Female	86	*SF3B1* H662Q	WT	RARS	0	Low	46, XX [29]
33	Male	84	*SF3B1* H662Q	WT	RARS	0	Low	45, X, −Y [17]/46, XY [8]
34	Male	69	WT	Exon 21	RA	0	Low	46, XY [25]

Supplementary Table S2: Significantly enriched predicted targets of the differentially expressed miRNAs relative to the remaining miRNAs ($P < 0.02$)

Gene Name	Putative oncogene or tumor suppressor*
HMGA1	Oncogene
CDV3	
TRAPPC1	
GYG2	
TRPM6	Oncogene
NHLRC3	
CYP19A1	Oncogene
SMIM3	Oncogene
ABCC5	Oncogene
CLDN12	Depending on cellular context
SOCS4	Tumor suppressor
KCNC2	
UTRN	Tumor suppressor
ERCC4	Tumor suppressor
IGDCC3	
CYP46A1	Oncogene
KLHDC8B	Tumor suppressor
COL1A2	
PBX2	Oncogene
SLC4A4	Oncogene
PLEKHH1	
ZNF10	
ARHGAP28	Oncogene
RPUSD3	
KLHL13	
FBXL12	
FASLG	Tumor suppressor
NDST2	
BIN3	Tumor suppressor
ACTA1	
NME6	
SCN11A	
RPUSD2	Oncogene
PLEKHO1	Tumor suppressor
PPP1R15B	Oncogene
QARS	Oncogene
BZW2	Oncogene
ZC3H3	
PLEKHG6	Oncogene
RGS16	Depending on cellular context
DLST	
ZNF318	

ADAMTS8	Depending on cellular context
ERGIC1	Oncogene
BACH1	Oncogene
TTLL4	Oncogene
RGS6	Tumor suppressor
COL14A1	
MEIS3	Oncogene
NPHP3	
ZBTB5	Oncogene
SLC20A1	Oncogene
GNG5	Oncogene
ATXN7L2	
ZNF583	
PLD3	
ERCC6	Tumor suppressor
LIPT2	
CHRD	Tumor suppressor
OSMR	Oncogene
ACSL6	Oncogene
LRIG3	
XK	

*Putative oncogene or tumor suppressor function was established following a Pubmed.gov search using "gene name" and "cancer" as search terms.

Supplementary Table S3: Associated KEGG pathways (adjusted *P* < 0.001) of the significantly enriched predicted targets

KEGG_ID	KEGG_PATHWAY
hsa00480	**Glutathione metabolism**
hsa03050	Proteasome
hsa00920	Sulfur metabolism
hsa04916	Melanogenesis
hsa04640	**Hematopoietic cell lineage**
hsa00410	**beta-Alanine metabolism**
hsa00250	**Alanine, aspartate and glutamate metabolism**
hsa03020	RNA polymerase
hsa00010	**Glycolysis/Gluconeogenesis**
hsa04650	Natural killer cell mediated cytotoxicity
hsa00130	Ubiquinone and other terpenoid-quinone biosynthesis
hsa00910	Nitrogen metabolism
hsa05310	Asthma
hsa05410	Hypertrophic cardiomyopathy (HCM)
hsa00140	Steroid hormone biosynthesis
hsa05120	Epithelial cell signaling in Helicobacter pylori infection
hsa00030	Pentose phosphate pathway
hsa04630	**Jak-STAT signaling pathway**

hsa00520	**Amino sugar and nucleotide sugar metabolism**
hsa00120	Primary bile acid biosynthesis
hsa00510	N-Glycan biosynthesis
hsa05221	**Acute myeloid leukemia**
hsa03018	RNA degradation
hsa05214	**Glioma**
hsa04512	ECM-receptor interaction
hsa04510	Focal adhesion
hsa04062	**Chemokine signaling pathway**
hsa04060	Cytokine-cytokine receptor interaction
hsa04210	**Apoptosis**
hsa05200	**Pathways in cancer**
hsa04660	T cell receptor signaling pathway
hsa03440	Homologous recombination
hsa03040	**Spliceosome**
hsa05016	Huntington's disease
hsa05010	Alzheimer's disease
hsa04722	Neurotrophin signaling pathway
hsa00790	Folate biosynthesis
hsa04114	Oocyte meiosis
hsa04614	Renin-angiotensin system
hsa05212	**Pancreatic cancer**
hsa05210	**Colorectal cancer**
hsa05215	**Prostate cancer**
hsa04664	Fc epsilon RI signaling pathway
hsa04666	Fc gamma R-mediated phagocytosis
hsa04920	Adipocytokine signaling pathway
hsa04010	**MAPK signaling pathway**
hsa04710	Circadian rhythm
hsa05332	Graft-versus-host disease
hsa00150	Androgen and estrogen metabolism
hsa04140	Regulation of autophagy
hsa05412	Arrhythmogenic right ventricular cardiomyopathy (ARVC)
hsa00020	**Citrate cycle (TCA cycle)**
hsa04130	SNARE interactions in vesicular transport
hsa05012	Parkinson's disease
hsa05330	Allograft rejection
hsa02010	ABC transporters
hsa04115	**p53 signaling pathway**
hsa05211	**Renal cell carcinoma**
hsa04514	Cell adhesion molecules (CAMs)
hsa04940	Type I diabetes mellitus
hsa04070	Phosphatidylinositol signaling system
hsa04930	Type II diabetes mellitus

Pathways discussed in the main part of the article are highlighted in bold.

Supplementary Table S4: Primer sequences for HRM and DGGE analyses

Gene (Hotspot)	Method	Primer sequence (5′-3′)
***SF3B1* (R625)**	HRM	Forward: CATGACTGTCCTTTCTTTGTTTACA<br>Reverse: GCAGAGGCTACAACAGCAAAAG
	DGGE	Forward: **CGCCCGCCGCGCCCCGCGCCCGTCCCGCCGCCCCCGCCCG** CATGACTGTCCTTTCTTTGTTTAC<br>Reverse: **CCCGCCCG**GCAGAGGCTACAACAGCAAAAG
***SF3B1* (H662)**	HRM	Forward: TCTTTATTGCCCTTCTTAAAAGC<br>Reverse: AGACAAAGTTACATTACAACTTAC
	DGGE	Forward: TCTTTATTGCCCTTCTTAAAAGC<br>Reverse:**CGCCCGCCGCGCCCCGCGCCCGTCCCGCCGCCCCCGCCCG** AGACAAAGTTACATTACAACTTAC
***SF3B1* (K700)**	HRM	Forward: AGTTAAAACCTGTGTTTGGTTTTGT<br>Reverse: CAAAAGATTCGATACCATAAGGAGT
	DGGE	Forward: **CGCCCGCCGCGCCCCGCGCCCGTCCCGCCGCCCCCGCCCG** AGTTAAAACCTGTGTTTGGTTTTGT<br>Reverse: **CCCGCCCGGC**AGAGGCTACAACAGCAAAAG
***U2AF35* (S34)**	HRM	Forward: AGTTAAAACCTGTGTTTGGTTTTGT<br>Reverse: CAAAAGATTCGATACCATAAGGAGT
***U2AF35* (Q157)**	HRM	Forward: ACCCGTGACGGACTTCAGAGA<br>Reverse: ACTGGCCACTCCTCACTCAC
***SRSF2* (P95)**	HRM	Forward: GCGAGCTGCGGGTGCAAATG<br>Reverse: CGGCGGCTGTGGTGTGAGT
***DNMT3A* (Exon 20)**	HRM	Forward: CTCCTTGGCTCATCTTCAAAC<br>Reverse: AGGCCCAGGAGCTTTCAC
***DNMT3A* (Exon 21)**	HRM	Forward: CTGTTATCCAGGTTTCTGTTGTTAC<br>Reverse: CCCAGCAGAGGTTCTAGACG
***DNMT3A* (Exon 22)**	HRM	Forward: CTTATTCCTCTTTTCTCCTCTTCA<br>Reverse: CAGATGCCAGCACAACCC

CG-tails are in bold.

Supplementary Table S5: Details of the miRNAs studied by RT-qPCR.

miRNA name	miRNA class	Taqman Assay ID	Genomic location (chromosome)	Host/ Overlapping Gene	Direction	Up-/down regulated in MDS
let-7a-5p	Canonical miRNA	000377				↓[1]
let-7a-1			9	*JB153432*	Same	
let-7a-2			11	*MIR100HG*	Same	
let-7a-3			22	*MIRLET7BHG*	Same	
let-7b-5p	Canonical miRNA	002619	22	*MIRLET7BHG*	Same	↑[2]
let-7c-5p	Canonical miRNA	000379	21	*MIR99AHG*	Same	↑[3]
let-7g-5p	Canonical miRNA	002282	3	*WDR82*	Same	
let-7i-5p	Canonical miRNA	002221	12	Intergenic	N/A	↓[4] ↑[3]
let-7f-5p	Canonical miRNA	000382				↑[3]
let-7f-1			9	*JB153432*	Same	
let-7f-2			X	*HUWE1*	Same	
hsa-miR-1	Canonical miRNA	002222	20	*C20orf166*	Same	↑[5, 6]
hsa-miR-100-5p	Canonical miRNA	000437	11	*MIR100HG*	Same	↑[3]
hsa-miR-106b-3p	Canonical miRNA	002380	7	*MCM7*	Same	↑[3]
hsa-miR-125b-5p	Canonical miRNA	000449				↑[7, 8]
hsa-miR-125b-1			11	*BC089451*	Same	
			11	*MIR100HG*	Same	
			11	*BX647608*	Same	
			11	*AK123947*	Same	
hsa-miR-125b-2			21	*MIR99AHG*	Same	
hsa-miR-126-3p	Canonical miRNA	002228	9	*EGFL7*	Same	↑[8, 9]
hsa-miR-130a-3p	Canonical miRNA	000454	11	*AK096335*	Same	↑[3, 8] ↓[10]
hsa-miR-132-3p	Canonical miRNA	000457	17	Intergenic	N/A	↓[10]
hsa-miR-10b-5p	Canonical miRNA	002218	2	Intergenic	N/A	↑[8, 9, 11]
hsa-miR-145-5p	Canonical miRNA	002278	5	*MIR143HG*	Same	↓[12-14]
hsa-miR-146a-5p	Canonical miRNA	000468	5	*DQ658414*	Same	↓[3, 13, 14]
hsa-miR-15a-5p	Canonical miRNA	000389	13	*DLEU2*	Same	↓[4] ↑[3, 9]
hsa-miR-181a-5p	Canonical miRNA	000480				↓[10, 15] ↑[9]
hsa-miR-181a-1			1	*MIR181AHG*	Same	
hsa-miR-181a-2			9	*NR6A1*	Opposite	
			9	*MIR181A2HG*	Same	
hsa-miR-10a-5p	Canonical miRNA	000387	17	*HOXB3*	Same	↑[3, 8, 9, 11]
hsa-miR-20a-5p	Canonical miRNA	000580	13	*MIR17HG*	Same	
hsa-miR-20b-5p	Canonical miRNA	001014	X	Intergenic	N/A	↑[3]
hsa-miR-23b-3p	Canonical miRNA	000400	9	*C9orf3*	Same	
hsa-miR-26b-5p	Canonical miRNA	000407	2	*CTDSP1*	Same	
hsa-miR-27b-3p	Canonical miRNA	000409	9	*C9orf3*	Same	↓[4]
hsa-miR-30c-5p	Canonical miRNA	000419				↓[10]
hsa-miR-30c-1			1	*NFYC*	Same	
hsa-miR-30c-2			6	Intergenic	N/A	
hsa-miR-34a-5p	Canonical miRNA	000425	1	*LOC106614088*	Same	↑[3-5, 8]
hsa-miR-378a-3p	Canonical miRNA	001314	5	*PPARGC1B*	Same	↓[4, 5, 16, 17]

hsa-miR-483-5p	Canonical miRNA	002338	11	*INS-IGF2*	Same	↓[16, 17]
			11	*IGF2*	Same	
hsa-miR-335-5p	Canonical miRNA	000546	7	*MEST*	Same	↑[8] ↓[10]
hsa-miR-486-5p	Canonical miRNA	001278	8	*ANK1*	Same	↑[8]
			8	*NKX6-3*	Same	
hsa-miR-130b-3p	Canonical miRNA	000456	22	Intergenic	N/A	↓[10]
hsa-miR-320a	Canonical miRNA	002277	8	Intergenic	N/A	↑[3, 18] ↓[10]
hsa-miR-124-3p	Canonical miRNA	001182				↓[3]
hsa-miR-124-1			8	Intergenic	N/A	
hsa-miR-124-2			8	*MIR124-2HG*	Same	
			8	*BX537900*	Same	
hsa-miR-124-3			20	Intergenic	N/A	
hsa-miR-326	Canonical miRNA	000542	11	*ARRB1*	Same	↓[3]
hsa-miR-149-3p	Canonical miRNA	002903	2	*GPC1*	Same	
			2	*PP14571*	Opposite	
hsa-miR-192-5p	Canonical miRNA	000491	11	*AB429224*	Same	
hsa-miR-140-3p	Canonical miRNA	002234	16	*WWP2*	Same	↑[17] ↓[10, 16]
hsa-miR-31-5p	Canonical miRNA	002279	9	*MIR31HG*	Same	
hsa-miR-18b-5p	Canonical miRNA	002217	X	Intergenic	N/A	
hsa-miR-203a	Canonical miRNA	000507	14	Intergenic	N/A	
hsa-miR-1275	Canonical miRNA	002840	6	Intergenic	N/A	
hsa-miR-941	Canonical miRNA	002183				
hsa-miR-941-1			20	*DNAJC*	Same	
hsa-miR-941-2			20	*DNAJC*	Same	
hsa-miR-941-3			20	*DNAJC*	Same	
hsa-miR-941-4			20	*DNAJC*	Same	
hsa-miR-941-5			20	*DNAJC*	Same	
hsa-miR-93-5p	Canonical miRNA	001090	7	*MCM7*	Same	
hsa-miR-186-5p	Canonical miRNA	002285	1	*ZRANB2*	Same	↑[3]
hsa-miR-95-3p	Canonical miRNA	000433	4	*ABLIM2*	Same	↓[8]
hsa-miR-15b-3p	Canonical miRNA	002173	3	*SMC4*	Same	
			3	*IFT80*	Opposite	
hsa-miR-18a-5p	Canonical miRNA	002422	13	*MIR17HG*	Same	↑[9]
hsa-miR-148a	Canonical miRNA	000470	7	Intergenic	N/A	↑[8]
hsa-miR-9-5p	Canonical miRNA	000583				
hsa-miR-9-1			1	*C1orf61*	Same	
hsa-miR-9-2			5	*LINC00461*	Same	
hsa-miR-9-3			15	*LINC00925*	Same	
hsa-miR-29a-3p	Canonical miRNA	002112	7	*LOC646329*	Same	
hsa-miR-329-3p	Canonical miRNA	001101				↑[5]
miR-329-1			14	Intergenic	N/A	
miR-329-2			14	Intergenic	N/A	
hsa-miR-107	Canonical miRNA	000443	10	*PANK1*	Same	
hsa-miR-191-5p	Canonical miRNA	002299	3	*DALRD3*	Same	↑[3]
			3	*NDUFAF3*	Opposite	

hsa-miR-423-5p	Canonical miRNA	002340	17	*NSPR1*	Same	↓[3, 5]
hsa-miR-103a-3p	Canonical miRNA	000439				↓[16] ↑[17]
hsa-miR-103a-1			5	*PANK3*	Same	
hsa-miR-103a-2			20	*PANK2*	Same	
hsa-miR-34b-3p	3′ SO miRNA *,†	002102	11	*BC021736*	Same	
hsa-miR-133a-3p	3′ SO miRNA*,†	002246				↑[6]
hsa-miR-133a-1			18	*miR133A1HG*	Same	
			18	*MIB1*	Opposite	
hsa-miR-133a-2			20	*MIR1-1HG*	Same	
hsa-miR-205-5p	3′ SO miRNA*,†	000509	1	*MIR205HG*	Same	↑[3]
hsa-miR-1287-5p	3′ SO miRNA*,†	002828	10	*PYROXD2*	Same	
hsa-miR-1292	3′ SO miRNA*,†	002824	20	*NOP56*	Same	
hsa-miR-302b-3p	SO miRNA on EST*	000531	4	*LARP7*	Same	
hsa-miR-627-5p	SO miRNA on EST*	001560	15	*VPS39*	Same	
hsa-miR-1248	SO miRNA on EST*	002870	3	*EIFA2*	Same	
			3	*SNORA81*	Same	
hsa-miR-3614	SO miRNA on EST*	461775_mat	17	*TRIM25*	Same	
			17	*BC114339*	Opposite	
hsa-miR-1204	5′ SO miRNA*,†	002872	8	*MYC*	Same	
			8	*PVT1*	Same	
hsa-miR-937-3p	3′→5′ SO miRNA*	002180	8	*SCRIB*	Same	
hsa-miR-1226-3p	Mirtron‡	245467_mat	3	*DHX30*	Same	
hsa-miR-3605-5p	5p tailed mirtron‡	463752_mat	1	*PHC2*	Same	
hsa-miR-4742-5p	5p tailed mirtron‡	463053_mat	1	*WDR26*	Same	
hsa-miR-3064-5p	5p tailed mirtron‡	465114_mat	17	*DDX5*	Same	
hsa-miR-6805-5p	5p tailed mirtron‡	466849_mat	19	*RPL28*	Same	
hsa-miR-4728-5p	5p tailed mirtron‡	461811_mat	17	*ERBB2*	Same	
hsa-miR-6515-5p	5p tailed mirtron‡	473040_mat	19	*CALR*	Same	
hsa-miR-5010-5p	5p tailed mirtron‡	476643_mat	17	*ATP6V0A1*	Same	
hsa-miR-4688	3p tailed mirtron‡	464507_mat	11	*DGKZ*	Same	
SNORD44	ncRNA	001094	1	*GAS5*	Same	N/A
RNU6B	ncRNA	001093	10	Intergenic	N/A	N/A
SNORD47	ncRNA	001223	1	*GAS5*	Same	N/A

Information was retrieved using the UCSC Genome Browser in September 2015 with the following tracks displayed: UCSC Genes, RefSeq Genes, sno/miRNA, and Spliced ESTs. References for the column "up-/down regulated in MDS" were retrieved following a literature search

*Mattioli C. *et al.*Cross talk between spliceosome and microprocessor defines the fate of pre-mRNA. Wiley Interdiscip Rev RNA. 2014 Sep-Oct; 5:647–58.

†Mattioli C. *et al*. A competitive regulatory mechanism discriminates between juxtaposed splice sites and pri-miRNA structures. 2013, Nucleic Acids Res. 2013 Oct; 41:8680–91.

‡miRNEST 2.0 (September 2015) (http://rhesus.amu.edu.pl/mirnest/copy/mirtrons_Ladewig.php).

REFERENCES

1. Zuo Z, Calin GA, de Paula HM, Medeiros LJ, Fernandez MH, Shimizu M, Garcia-Manero G, Bueso-Ramos CE. Circulating microRNAs let-7a and miR-16 predict progression-free survival and overall survival in patients with myelodysplastic syndrome. Blood. 2011; 118:413–415.
2. Karoopongse E, Yeung C, Byon J, Ramakrishnan A, Holman ZJ, Jiang PY, Yu Q, Deeg HJ, Marcondes AM. The KDM2B- let-7b -EZH2 axis in myelodysplastic syndromes as a target for combined epigenetic therapy. PLoS One. 2014; 9:e107817.
3. Sokol L, Caceres G, Volinia S, Alder H, Nuovo GJ, Liu CG, McGraw K, Clark JA, Sigua CA, Chen DT, Moscinski L, Croce CM, List AF. Identification of a risk dependent microRNA expression signature in myelodysplastic syndromes. Br J Haematol. 2011; 153:24–32.
4. Merkerova MD, Krejcik Z, Belickova M, Hrustincova A, Klema J, Stara E, Zemanova Z, Michalova K, Cermak J, Jonasova A. Genome-wide miRNA profiling in myelodysplastic syndrome with del(5q) treated with lenalidomide. Eur J Haematol. 2015; 95:35–43.
5. Dostalova Merkerova M, Krejcik Z, Votavova H, Belickova M, Vasikova A and Cermak J. Distinctive microRNA expression profiles in CD34+ bone marrow cells from patients with myelodysplastic syndrome. Eur J Hum Genet. 2011; 19:313–319.
6. Hussein K, Theophile K, Busche G, Schlegelberger B, Gohring G, Kreipe H and Bock O. Aberrant microRNA expression pattern in myelodysplastic bone marrow cells. Leuk Res. 2010; 34:1169–1174.
7. Bousquet M, Quelen C, Rosati R, Mansat-De Mas V, La Starza R, Bastard C, Lippert E, Talmant P, Lafage-Pochitaloff M, Leroux D, Gervais C, Viguie F, Lai JL, et al. Myeloid cell differentiation arrest by miR-125b-1 in myelodysplastic syndrome and acute myeloid leukemia with the t(2;11)(p21;q23) translocation. J Exp Med. 2008; 205:2499–2506.
8. Votavova H, Grmanova M, Dostalova Merkerova M, Belickova M, Vasikova A, Neuwirtova R, Cermak J. Differential expression of microRNAs in CD34+ cells of 5q- syndrome. J Hematol Oncol. 2011; 4:1.
9. Pons A, Nomdedeu B, Navarro A, Gaya A, Gel B, Diaz T, Valera S, Rozman M, Belkaid M, Montserrat E, Monzo M. Hematopoiesis-related microRNA expression in myelodysplastic syndromes. Leuk Lymphoma. 2009; 50:1854–1859.
10. Maki K, Sasaki K, Nagata Y, Nagasawa F, Nakamura Y, Ogawa S, Mitani K. Expressional changes of genes and miRNA in common megakaryocyte-erythroid progenitors from lower-risk myelodysplastic syndrome. Int J Hematol. 2014; 100:361–369.
11. Li X, Xu F, Chang C, Byon J, Papayannopoulou T, Deeg HJ, Marcondes AM. Transcriptional regulation of miR-10a/b by TWIST-1 in myelodysplastic syndromes. Haematologica. 2013; 98:414–419.
12. Kumar MS, Narla A, Nonami A, Mullally A, Dimitrova N, Ball B, McAuley JR, Poveromo L, Kutok JL, Galili N, Raza A, Attar E, Gilliland DG, Jacks T and Ebert BL. Coordinate loss of a microRNA and protein-coding gene cooperate in the pathogenesis of 5q- syndrome. Blood. 2011; 118:4666–4673.
13. Oliva EN, Cuzzola M, Aloe Spiriti MA, Poloni A, Lagana C, Rigolino C, Morabito F, Galimberti S, Ghio R, Cortelezzi A, Palumbo GA, Sanpaolo G, Finelli C, et al. Biological activity of lenalidomide in myelodysplastic syndromes with del5q: results of gene expression profiling from a multicenter phase II study. Ann Hematol. 2013; 92:25–32.
14. Starczynowski DT, Kuchenbauer F, Argiropoulos B, Sung S, Morin R, Muranyi A, Hirst M, Hogge D, Marra M, Wells RA, Buckstein R, Lam W, Humphries RK, et al. Identification of miR-145 and miR-146a as mediators of the 5q- syndrome phenotype. Nat Med. 2010; 16:49–58.
15. Santamaria C, Muntion S, Roson B, Blanco B, Lopez-Villar O, Carrancio S, Sanchez-Guijo FM, Diez-Campelo M, Alvarez-Fernandez S, Sarasquete ME, de las Rivas J, Gonzalez M, San Miguel JF, et al. Impaired expression of DICER, DROSHA, SBDS and some microRNAs in mesenchymal stromal cells from myelodysplastic syndrome patients. Haematologica. 2012; 97:1218–1224.
16. Erdogan B, Facey C, Qualtieri J, Tedesco J, Rinker E, Isett RB, Tobias J, Baldwin DA, Thompson JE, Carroll M, Kim AS. Diagnostic microRNAs in myelodysplastic syndrome. Exp Hematol. 2011; 39:915–926 e912.
17. Erdogan B, Bosompem A, Peng D, Han L, Smith E, Kennedy ME, Alford CE, Wu H, Zhao Z, Mosse CA, El-Rifai W, Kim AS. Methylation of promoters of microRNAs and their host genes in myelodysplastic syndromes. Leuk Lymphoma. 2013; 54:2720–2727.
18. Choi JS, Nam MH, Yoon SY, Kang SH. MicroRNA-194-5p could serve as a diagnostic and prognostic biomarker in myelodysplastic syndromes. Leuk Res. 2015; 39:763–768.
